# Preparation, characterization and hydrogenation activity of multiply-reduced silicotungstic acid

**DOI:** 10.1039/d6ra02211a

**Published:** 2026-07-03

**Authors:** Ahmed Aboorh, Zeliha Ertekin, Sarah K. Dugmore, Claire Wilson, Stephen Sproules, Mark D. Symes

**Affiliations:** a School of Chemistry, University of Glasgow Glasgow G12 8QQ UK mark.symes@glasgow.ac.uk; b Department of Chemistry, College of Sciences, University of Jeddah Jeddah 23890 Saudi Arabia

## Abstract

Silicotungstic acid (H_4_SiW_12_O_40_) demonstrates some fascinating reversible redox behavior that in recent years has been exploited both for decoupled electrolysis and for the selective hydrogenation of nitroarenes to their aniline derivatives without the need to use hydrogen gas or precious metal catalysts. Specifically, silicotungstic acid can be reduced electrochemically to its two-electron reduced form (H_6_SiW_12_O_40_), which displays good stability and which can be induced to cycle multiple times between H_6_SiW_12_O_40_ and H_4_SiW_12_O_40_ on account of its reversible reduction and re-oxidation. In contrast, studies on more deeply-reduced states of silicotungstic acid are rarely addressed in the literature, although the potential for developing more powerful, yet still selective, hydrogenation agents if more deeply-reduced states could be accessed reliably offers an incentive to explore their formation. Herein, we report the controlled and reproducible formation of silicotungstic acid in its previously uncharacterized four-electron reduced form by judicious selection of electrochemical reduction parameters. We obtain and compare the crystal structures of two- and four-electron reduced silicotungstic acid for the first time and show that four-electron reduced silicotungstic acid is indeed a more effective hydrogenation agent for the reduction of 4-cyanobenzaldehyde and 4-(trifluoromethyl)benzaldehyde than the two-electron reduced form. Our results suggest that careful optimization of reduction conditions can allow polyoxometalates in oxidation states hitherto considered rather exotic to be produced reliably, and subsequently deployed to produce reaction outcomes that are demonstrably different from those occasioned by their less-reduced analogues.

## Abstract 2

Silicotungstic acid (H_4_SiW_12_O_40_) exhibits reversible redox behaviour exploited in decoupled electrolysis and selective hydrogenation of nitroarenes to anilines, avoiding hydrogen gas and precious metal catalysts.

## Graphical abstract

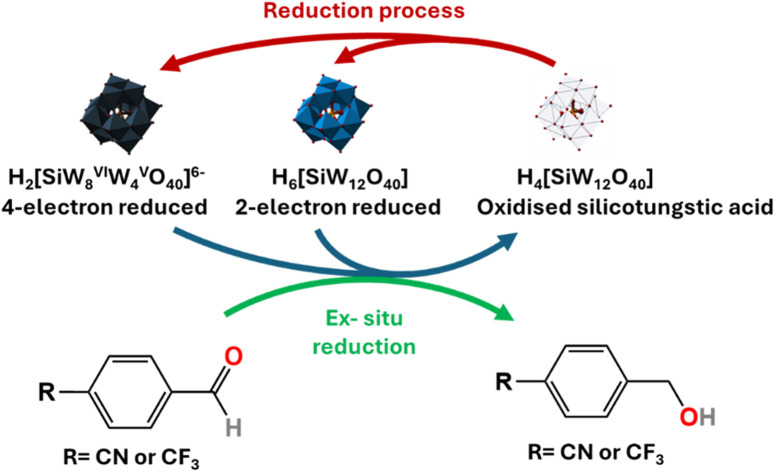

## 1 Introduction

The use of a reduced redox mediator as a solution-phase hydrogenation agent (an “indirect electrolysis” approach) offers a potential method for the selective hydrogenation of substrates under mild conditions.^1^ Among the various redox mediators explored to date, silicotungstic acid is a particularly promising candidate for mediating indirect electrochemical hydrogenation reactions. It belongs to the class of metal oxides known as the polyoxometalates, a family of molecular metal oxides distinguished by their ability to reversibly store and transfer multiple electrons. This property is often described as giving polyoxometalates “electron reservoir” or “electron sponge” behavior.^2,3^

Rather few studies have explored the reduction of silicotungstic acid beyond the two electron reduced state, primarily because deeper reduction requires more negative potentials, which can lead to the formation of highly reduced tungsten blue and brown species that are not readily re-oxidized to their original forms.^4^ Under such conditions, electrode surface modification and promotion of the hydrogen evolution reaction can also occur, further complicating controlled multi-electron reduction.^4,5^

Consequently, most studies involving silicotungstic acid have focused on the two-electron reduced state, which is relatively easy to obtain and whose formation is reversible.^6^ For example, MacDonald *et al.* reported that the two electron reduced form of the polyoxometalate silicotungstic acid can act as a selective and effective hydrogenation agent for the nitro group in various nitroarenes, producing the aniline derivatives in high yields without the need for co-catalysts whilst tolerating the presence of other functional groups.^7^

Careful control of both the applied potential and reduction time is critical to achieve multi-electron reduction without inducing side reactions. Hervé studied the electrochemical reduction of silicotungstate anion [α-SiW_12_O_40_]^4−^ in aqueous solution.^2,8^ When bulk electrolysis was used to target a four-electron-reduced species, a more deeply reduced, eight-electron species was instead formed. This dark blue solution exhibited a voltammogram markedly different from that of the original polyoxometalate, with oxidation waves corresponding to two- and six-electron processes. Subsequent oxidation of the dark blue species yielded a brown material.^2^ The nature of such brown reduced polyoxotungstate species varies depending on the oxidation states of the tungsten centres (W^4+^, W^5+^, and W^6+^).^8^ Irreversible electrode reactions observed at more negative potentials are attributed to the formation of W^4+^, stabilized through metal–metal bonding in the highly reduced species.^9^ These observations highlight the difficulty of isolating well-defined multi-electron reduced forms without triggering irreversible pathways.

Despite these challenges, achieving controlled multi-electron reduction is highly desirable, as increasing the stored electron count may substantially enhance the reducing efficiency and reactivity of the mediator. Herein, we report a controlled electrochemical approach that enables the reproducible and fully reversible formation of both two-electron- and four-electron-reduced silicotungstic acid species in aqueous conditions. Both the two-electron- and four-electron-reduced forms of silicotungstic acid were prepared and characterized using single-crystal X-ray diffraction. The four-electron-reduced species was generated without formation of brown precipitates by applying minimally negative potentials for extended durations to achieve the required depth of reduction, enabled by the anodic shift of the relevant redox waves as the reduction proceeds. This controlled approach allows direct examination of structural changes associated with the reduction of silicotungstic acid.

**Fig. 1:**
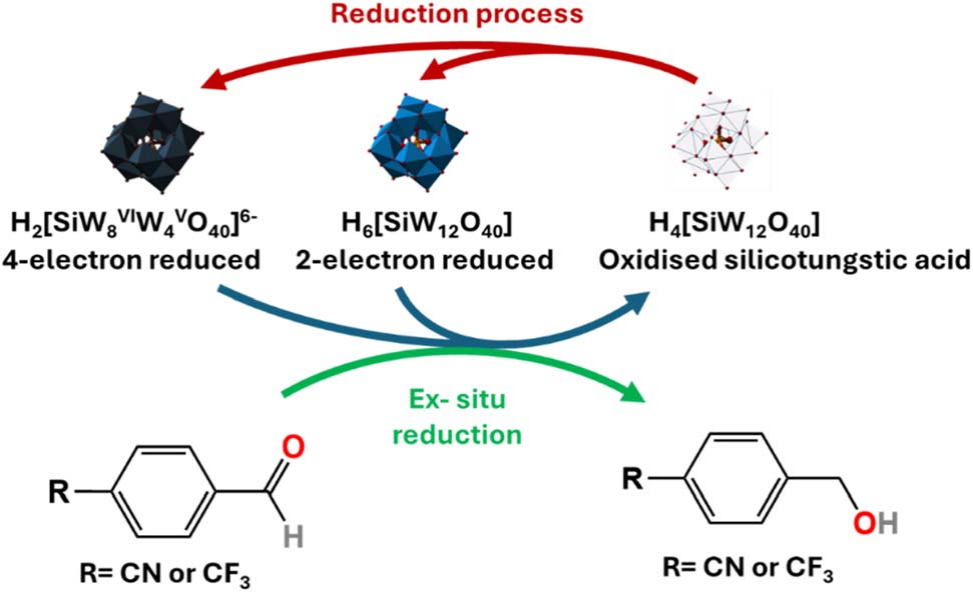
Proposed hydrogenation reaction of aryl aldehydes with electron withdrawing groups (–CN and –CF_3_) mediated by 2e^−^ or 4e^−^ reduced silicotungstic acid.

Furthermore, we hypothesized that redox mediators capable of accepting more electrons should act as more effective reducing agents. We therefore examined the catalyst-free hydrogenation of aryl aldehydes with cyano (–CN) and trifluoromethyl (–CF_3_) electron-withdrawing groups using silicotungstic acid in various states of reduction, as summarized in Fig. 1. Polyoxometalate clusters such as silicotungstic acid are well-established in the literature to display high water solubility and the ability to reversibly accept and donate multiple electrons and protons through outer-sphere mechanisms, without the formation of any bond between substrate and mediator; consequently, the hydrogenation reaction presumably proceeds spontaneously upon contact of the reduced heteropolyanion with the substrate, driven by the thermodynamic favourability dictated by their relative formal reduction potentials.^1,10^ The results highlight that the depth of reduction of the polyoxometalate redox mediator does indeed influence the efficacy of reduction (for example, increasing the yield of 4-(hydroxymethyl)benzonitrile from the reduction of 4-cyanobenzaldehyde from 43% using two-electron reduced silicotungstic acid to 81% using four-electron reduced silicotungstic acid under otherwise identical conditions). These results in turn suggest that controlled electrochemical reduction of other multi-electron accepting redox mediators could be a fruitful avenue for generating mediators with enhanced properties for specific chemical conversions.

## 2 Experimental

### 2.1 Materials

Tungstosilicic acid hydrate, (H_4_[Si(W_3_O_10_)_4_]·*x*H_2_O, reagent grade, CAS: 12 027-43-9) and phosphoric acid (H_3_PO_4_, 85% aqueous solution, CAS: 7664-38-2) were purchased from Thermo Fisher. 4-Cyanobenzaldehyde (C_8_H_5_NO, 98+%, CAS: 105-07-7), 4-(trifluoromethyl) benzaldehyde (CF_3_C_6_H_4_CHO, 98%, CAS: 455-19-6), and phthalonitrile (C_6_H_4_(CN)_2_, 98%, CAS: 91-15-6) were obtained from Thermo Scientific. Terephthalonitrile (C_8_H_4_N_2_, 98%, CAS: 623-26-7), 4-(trifluoromethyl) acetophenone (CF_3_C_6_H_4_COCH_3_, CAS: 709-63-7), and activated carbon (CAS:7440-44-0) were sourced from Alfa Aesar. Biphenyl-4-carboxaldehyde (C_6_H_5_C_6_H_4_CHO, CAS: 3218-36-8), 2-naphthaldehyde (C_10_H_7_CHO, CAS: 66-99-9), and diethyl ether (≥99.8%, CAS:60-29-7) were acquired from Sigma-Aldrich and used as received. Sodium hydroxide (NaOH, CAS: 1310-73-2), magnesium sulfate (dried, ≥ 98%, MgSO_4_, CAS: 7487-88-9) and hydrochloric acid (HCl, CAS: 7647-01-0) were purchased from Honeywell and used as received. Chloroform (CHCl_3_, 99.8+, certified AR for analysis, stabilized with amylene, CAS: 67-66-3) was obtained from Fisher Scientific. All chemicals were used without further purification. Nafion membrane 117 was obtained from fuel cell store and stored in 1.0 M H_2_SO_4_. Nitrogen (>99% purity) was purchased from BOC Ltd. Chloroform-d, DLM-7-100 was obtained from Cambridge Isotope Laboratories (CDCl_3_, CAS: 865-49-6).

### 2.2 Electrochemical set-up

Cyclic voltammetry (CV) experiments were performed using a three-electrode setup in single-chamber cells at room temperature (∼20 °C). The measurements were conducted at a scan rate of 100 mV s^−1^ using a Gamry Interface 1010E, without stirring and without *iR* compensation. The solution (10 mL) was thoroughly degassed with nitrogen for 5 minutes before measurements were collected and was kept under an inert atmosphere throughout the process. A glassy carbon disc electrode (surface area = 0.071 cm^2^) served as the working electrode, a platinum wire was used as the counter electrode, and an Ag/AgCl (3 M NaCl) reference electrode was employed.

The bulk electrolysis experiments were conducted using a three-electrode configuration in a two-compartment H-cell. The compartments were separated by a Nafion N-117 membrane to permit H^+^ ion diffusion between the electrolytes. The cathode compartment contained a 0.5 M aqueous silicotungstic acid solution with a carbon felt working electrode (total geometric dimensions: 2.5 × 4.0 cm^2^) and an Ag/AgCl reference electrode, while the anode compartment contained 1.0 M aqueous H_3_PO_4_ (pH = ∼0.5) and a platinized titanium counter electrode. The cathode compartment was stirred at around 300 rpm, and both compartments were continuously bubbled with nitrogen/argon to avoid oxidation of the solution by the air. Bulk electrolysis was then carried out at −0.36 V *vs.* NHE to selectively generate the initial 2-electron reduced form of silicotungstic acid, as evidenced by the current declining to background levels of less than 20 mA after about 16 hours. After preparing the 2e^−^ reduced solution of silicotungstic acid, it was removed from the working electrode compartment of the H-cell.

**Fig. 2:**
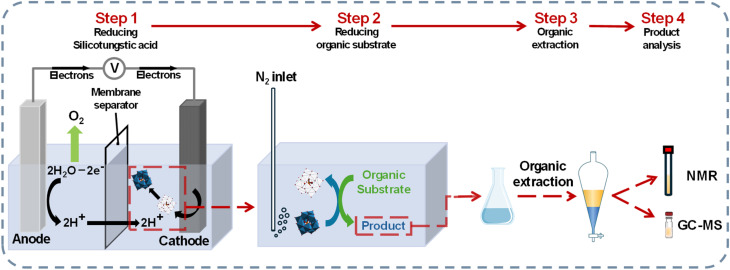
An overview of the hydrogenation process for organic substrates using reduced silicotungstic acid: Step 1 involves the electrochemical reduction of silicotungstic acid (oxidised form shown in white, reduced form shown in blue). In Step 2, the reduced silicotungstic acid hydrogenates the organic substrates. Step 3 involves the extraction of the products. Finally, Step 4 consists of analysing the products using ^1^H NMR and GC-MS.

Organic compounds were subsequently added to the reduced silicotungstic acid solution in ratios of either 1 : 1 or 1 : 10, corresponding to 1 part organic compounds to 1 or 10 parts silicotungstic acid. These mixtures were stirred using a magnetic stirrer in a round-bottom flask and allowed to react overnight (approximately 18 hours) under a nitrogen atmosphere at 20 °C. The nitrogen atmosphere was maintained by continuously flowing nitrogen gas over the reaction mixture through a gas inlet and outlet system. Following reaction, the pH of the acidic solution was adjusted to approximately 7 using 1.0 M NaOH to neutralize the strongly acidic medium and facilitate efficient extraction of the neutral benzylic alcohol products into the organic phase in their neutral forms. The mixture was then extracted with chloroform in two 30 mL aliquots. The organic phase was subsequently dried over MgSO_4_, filtered through filter paper, and concentrated using a rotary evaporator. This process is summarized in Fig. 2. Reduced silicotungstic acid was recycled following a literature method previously reported in detail by our group.^7^ Recycled silicotungstic acid was reused as a hydrogenation agent in reactions with organic substrates, with adjustment of substrate loading where required. The recovered aqueous silicotungstic acid solution was partially evaporated, acidified with concentrated HCl to pH < 1, and heated at 95 °C for 24 h to regenerate the Keggin structure. After activated carbon treatment and filtration, silicotungstic acid was separated from the mother liquor by diethyl ether/HCl extraction and concentrated by rotary evaporation to afford a solid, which was repeatedly dissolved and re-evaporated to remove residual HCl before recrystallisation and drying at 100 °C overnight. The regenerated silicotungstic acid was then electrochemically reduced, after which organic substrates were added to the reduced silicotungstic acid solution to repeat the hydrogenation process. The extraction and reuse cycle was repeated three times.

### 2.3 Analysis of reduced organic substrates

To analyse the products, nuclear magnetic resonance (NMR) spectroscopy was employed. ^1^H NMR spectra were recorded at room temperature using a Bruker 400 MHz NMR spectrometer. The products were dissolved in CDCl_3_ for this analysis. An Agilent GC-MS instrument equipped with a 7890A gas chromatograph and a 5975C inert mass selective detector (triple-axis detector), and an Agilent J&W DB-35 ms column (30 m × 0.32 mm, 0.25 µm: Part Number: 123-3832) was used for further analysis of the obtained products. All obtained products were dissolved in dichloromethane before analysis. The initial oven temperature was set to 45 °C and held for 3 minutes. It was then ramped at a rate of 20 °C per minute until reaching 275 °C and held for 5 minutes. The total analysis time was 19.5 minutes. Ultraviolet-visible (UV-vis) spectroscopy was also employed to analyse both liquid and solid samples of reduced silicotungstic acid. Liquid sample measurements were performed using an Agilent Cary 60 UV-vis spectrophotometer (wavelength range: 190–1100 nm), while solid sample measurements were carried out using a Shimadzu UV-2600 spectrophotometer (wavelength range: 185–900 nm) equipped with an air sensitive solid sample holder. Single-crystal X-ray diffraction data were collected for 2e^−^ reduced and 4e^−^ reduced silicotungstic acid at 150 K on a Rigaku XtaLAB Synergy R diffractometer, using a HyPix-Arc 150 detector, equipped with graphite-monochromated Mo Kα radiation (*λ* = 0.71073 Å) micro-focus sealed X-ray source (50 kV, 24 mA). Data collection and reduction was performed using CrysAlisPro software package, and structures were solved and refined using Olex 1.5. For oxidized silicotungstic acid, single-crystal X-ray diffraction data was obtained at 150 K using Bruker D8 Venture with a Photon II detector, Mo Kα radiation (*λ* = 0.71073 Å) micro-focus sealed X-ray source (50 kV, 24 mA) at 150 K. Data collection and reduction was performed using APEX4 and SAINT V8.37A. To evaluate the reusability of silicotungstic acid, oxidized silicotungstic acid, and their two-electron and four-electron reduced forms, the structures of the recycled materials were characterised using Fourier Transform Infrared (FTIR) spectroscopy. FTIR spectra were recorded using a Nicolet™ Summit™ FTIR spectrometer.

## 3 Results and discussion

### 3.1 Reduction of silicotungstic acid

**Fig. 3:**
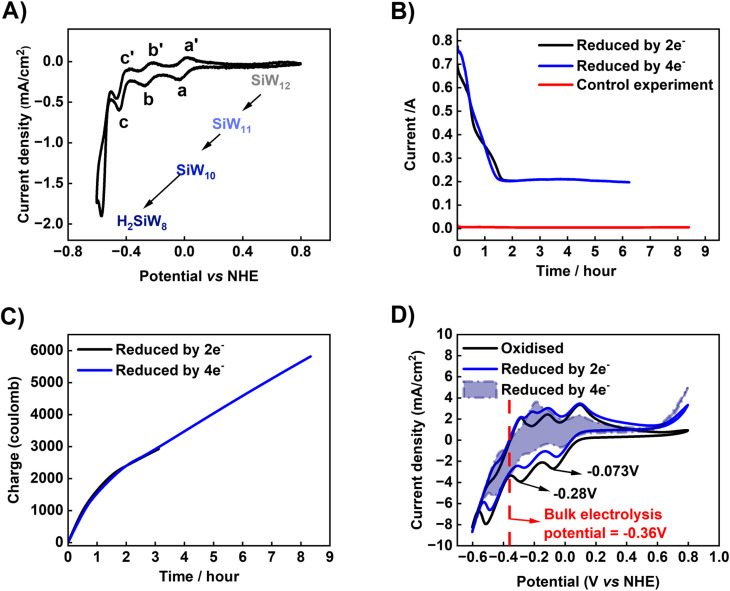
(A) Cyclic voltammogram of 50 mM silicotungstic acid solution recorded with a glassy carbon working electrode (0.071 cm^2^), a platinum wire counter electrode, and an Ag/AgCl reference electrode, at a scan rate of 10 mV s^−1^, within a potential range of 0.8 to −0.6 V *vs.* NHE. The following species are indicated: SiW_12_ (SiW_12_^VI^O_40_^4−^, oxidized), SiW_11_ (SiW_11_^VI^W^V^O_40_^5−^, reduced by one electron), SiW_10_ (SiW_10_^VI^W_2_^V^O_40_^6−^, reduced by two electrons), and H_2_SiW_8_ (H_2_[SiW_8_^VI^W_4_^V^O_40_]^6−^, reduced by four electrons). (B) Current–time plots for controlled potential electrolyses in an H-cell at −0.36 V *vs.* NHE: a 2-electron reduction of 30 mL of 0.5 M silicotungstic acid (black line), a 4-electron reduction under the same conditions (blue line), and a control using just 1 M phosphoric acid (red line), and (C) the corresponding charge-time plots for the data in panel B. The charge required for a 2-electron reduction of this amount of silicotungstic acid is 2895 C, and for complete 4-electron reduction 5789 C is required. (D) Cyclic voltammograms of 0.5 M silicotungstic acid recorded in its initial (fully oxidized) state, after a 2-electron reduction, and after a 4-electron reduction, using the same electrode configuration as in panel A and a scan rate of 100 mV s^−1^.

The cyclic voltammogram of 50 mM silicotungstic acid in Fig. 3(a) displays three pairs of redox waves with formal potentials at −0.043 V, −0.273 V, and −0.450 V *vs.* NHE. These correspond to two successive one-electron transfer processes, followed by a two electron transfer process at −0.450 V.^11,12^ The corresponding cathodic and anodic peaks indicate that all three redox processes are electrochemically reversible, meaning the reduced species can be re-oxidized back to their original states.^5,12,13^

The three main redox processes for silicotungstic acid can be summarised as follows:^14^

1 SiW_12_^VI^O_40_^4−^ + e^−^ ⇌ SiW_11_^VI^W^V^O_40_^5−^

2 SiW_11_^VI^W^V^O_40_^5−^ + e^−^ ⇌ SiW_10_^VI^W_2_^V^O_40_^6−^

3 SiW_10_^VI^W_2_^V^O_40_^6−^ + 2e^−^ + 2H^+^ ⇌ H_2_[SiW_8_^VI^W_4_^V^O_40_]^6−^

Eqn (1) is the one-electron reduction of silicotungstic acid, seen as the first cathodic peak at −0.043 V *vs.* NHE in the cyclic voltammogram (Fig. 3(a)). A single electron is added to the SiW_12_^VI^O_40_^4−^ species, generating SiW_11_^VI^W^V^O_40_^5−^. This reduction process causes a change in colour, and the solution turns blue. Eqn (2) shows the second one-electron reduction of silicotungstic acid, resulting in SiW_10_^VI^W_2_^V^O_40_^6−^, and the solution becomes more intensely blue as the reduction continues. Eqn (3) involves the transfer of two electrons and the addition of two protons to the already two-electron reduced species, resulting in H_2_[SiW_8_^VI^W_4_^V^O_40_]^6−^. The solution becomes even darker blue as electron transfer continues, due to the appearance of intervalence charge transfer bands.^15,16^ As shown in Fig. S1, the colour change of the solution is reversible, with the solution returning to colourless upon complete reoxidation.

During bulk electrolysis for both the 2-electron (black line) and 4-electron (blue line) reduction processes (Fig. 3(b)), the current does not drop to zero. In contrast, the control experiment without any mediator (red line), shows a very low current, indicating minimal background activity. This observation does not indicate a background process for the blue and black lines but instead arises from continued reduction of silicotungstic acid. The charge–time plots in Fig. S2, showing reversible 2-electron and 4-electron reduction and reoxidation of silicotungstic acid, further support this interpretation. The measured charge closely matches the theoretical value required for the transfer of 2-electrons and 4-electrons, and the reduced species can be fully re-oxidised, demonstrating that both the 2-electron and 4-electron reduced forms are generated reversibly without significant parasitic hydrogen evolution.

To investigate this reduction behaviour of silicotungstic acid in more detail, electrolysis was conducted at a constant potential of −0.36 V *vs.* NHE for 8 days, followed by an oxidation step at +0.76 V *vs.* NHE. The resulting charge–time profile is shown in Fig. S3, while the corresponding volume of hydrogen gas produced as a function of total charge passed is presented in Fig. S4. The data suggest that hydrogen evolution initiates after the transfer of approximately four electrons per silicotungstic acid molecule (see Fig. S5). Beyond this point, while most of the additional charge still contributes to further reduction of the polyoxometalate, hydrogen evolution becomes increasingly competitive as electrolysis continues. An additional experiment was performed at a fixed current of 300 mA (Fig. S6). Under these uncontrolled potential conditions, brown precipitates formed together with increased hydrogen evolution, indicating that controlled generation of the re-oxidizable dark blue 4-electron reduced species requires careful control of the potential to avoid over-reduction.

Beyond 4-electron reduction, reduced silicotungstic acid generates red-brown solutions that cannot be fully re-oxidized at a potential of +0.76 V (*vs.* NHE) (see Fig. S7). This response suggests the formation of species similar to those observed previously with highly-reduced metatungstate species.^7,17^ Dong *et al.* reported that the four electron reduced silicotungstic acid exhibits strong electrocatalytic activity for nitrite reduction under pH conditions below 2.^14^ However, their study focused primarily on electrocatalytic performance and did not include detailed characterization of the stability, reversibility, or broader reactivity of the four electron reduced species. Consequently, information on the four electron reduced state of silicotungstic acid remains limited in the literature, as most studies primarily focus on its two electron reduced form. Therefore, comparing the catalytic efficiency of the two electron reduced silicotungstic acid with its four electron reduced form remains of interest.

Fig. 3(c) shows the charge–time plot of silicotungstic acid, illustrating the 2-electron reduction and further reduction to the 4-electron-reduced form. The reduction behaviour of silicotungstic acid is influenced by the number of electrons transferred.^12,18^ To investigate this, cyclic voltammetry was performed on the two electron and four electron reduced forms of silicotungstic acid, and the results were compared with those of the oxidized form (Fig. 3(d)). The electrochemical responses of the reduced species exhibit some differences, with noticeable anodic shifts in their reduction peaks compared to the oxidized form, indicating that the redox behaviour is influenced by the number of electrons transferred during reduction. As a result, multi-electron reduction becomes somewhat more accessible as the polyoxometalate is reduced; by holding the potential at the relatively mild cathodic bias of −0.36 V *vs.* NHE, one is able to obtain a deeper state of reduction than might be assumed on the basis of the cyclic voltammogram of the fully oxidized form of silicotungstic acid.

UV-vis spectra of the two electron and four electron reduced forms are presented in Fig. S8(a). Although the spectra for the two species differ only slightly in solution, more pronounced differences are observable in the solid state UV-vis spectra (Fig. S8b), with absorption maxima at approximately 527.5 nm for the two electron reduced species and 549.6 nm for the four electron reduced species.

### 3.2 Single crystal structure measurements

Single-crystal X-ray diffraction (SCXRD) was used to examine any structural changes that may occur during the reduction of silicotungstic acid and to compare the reduced forms with the oxidized structure as reported in the literature.^19^ To obtain crystals suitable for SCXRD analysis, 1 mL of 0.5 M of reduced silicotungstic acid solution was prepared under nitrogen and transferred *via* syringe into 3 mL of 1.0 M KCl solution that had been degassed prior to mixing in a glass vessel fitted with a plastic cap. The vessel was then placed inside a plastic bag, purged with nitrogen, sealed to maintain an inert atmosphere, and stored in the fridge at 4 °C for 2 days to allow crystal growth.

The compounds [SiW_10_^VI^W_2_^V^O_40_]^6−^ (two electron reduced silicotungstic acid) and H_2_[SiW_8_^VI^W_4_^V^O_40_]^6−^ (four electron reduced silicotungstic acid) crystallise in a hexagonal space group *P*6_2_ 2 2 and retain the Keggin type framework. Bond valence calculations indicate an expected average tungsten oxidation state of +5.83 for the two electron reduced silicotungstic acid and +5.67 for the four electron reduced silicotungstic acid, consistent with the reduction of two and four W(vi) centres to W(v), respectively. Three crystallographically independent W sites are observed, which are generated by the symmetry operations in the space group. There are no statistically significant bond length variations between the reduced and oxidized structures, only slight contractions in the range as expected for the variation in temperature. Due to the 3 crystallographically independent W sites, it is necessary that there is averaging of the oxidation states across sites rather than clearly localised W(v) or W(vi) centres, this is due to the model being time averaged over the entirety of the data collection. The key crystallographic parameters for oxidized silicotungstic acid and both its reduced species are summarised in Table S1 (more detailed tables can be found in the SI, Tables S2–S7).

Both reduced structures were refined with partial potassium occupancies (∼2.7 K^+^ per anion) and the solvent content was modelled by a solvent mask (SQUEEZE) due to the disorder of the water in the channels which form parallel to the *c*-axis (Fig. S9 and S10); this accounts for around 4 water molecules per asymmetric unit. Ellipsoid plots showing partial occupancies at the 50% probability level of the asymmetric unit are provided in Fig. S11 and S12. Both reduced anions maintain the characteristic Keggin framework, with only minor differences in lattice parameters (Δ*a* ≈ 0.04 Å, Δ*V* ≈ 10 Å^3^), these small differences indicate that electron addition or proton removal mainly alters the electronic distribution within the cluster rather than its overall geometry. Although the two reduced forms do not display significant structural differences between them, a noticeable unit cell contraction is observed when compared to the oxidized silicotungstic acid. Overall, there is a contraction of around 0.78% between the oxidised and four electron-reduced lattice parameters (Δ*a* ≈ 0.045 Å, Δ*V* ≈ 31.85 Å^3^) at 150 K. The contraction upon cooling of the oxidised structure is ∼1.5% (Δ*V* ≈ 49.02 Å^3^). This demonstrates that the temperature variation between the published oxidised structure at 293 K and the two reduced structures published is not only a factor of temperature but also the state of reduction. Additionally, the reduction changes the compound from colourless to deep blue to almost black, with an increase in the size of the crystals formed as well.

### 3.3 Product analysis from substrates treated with reduced silicotungstic acid

Optimisation of the hydrogenation reactions was performed by varying the mole ratio of substrate to mediator and comparing the effects of the 2-electron and 4-electron reduced forms of the mediator. Substrate conversion was determined by integration of the relevant ^1^H NMR resonances, while product yields were determined from the isolated mass obtained after removal of the reaction solvent using a rotary evaporator (see Experimental Section 2.2), with molecular weights used to relate mass to molar amount.

**Fig. 4:**
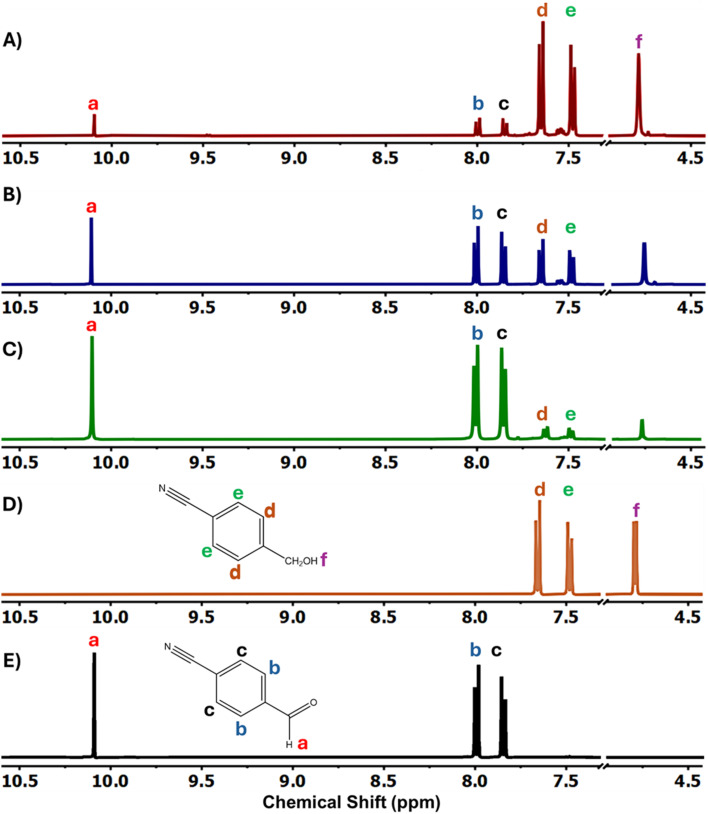
(A) Stacked ^1^H NMR spectrum of the reduction products obtained after extraction using 4-electron-reduced silicotungstic acid at a 10 : 1 ratio. Stacked ^1^H NMR spectra of the reduction products obtained after extraction using 2-electron-reduced silicotungstic acid at ratios of 10 : 1 (B) and 1 : 1 (C). ^1^H NMR spectrum of commercial 4-(hydroxymethyl)benzonitrile (D). ^1^H NMR spectrum of the starting material, 4-cyanobenzaldehyde (E).

In Fig. 4, stacked ^1^H NMR spectra of the reduction of 4-cyanobenzaldehyde using reduced silicotungstic acid as the hydrogenation agent are presented (a ^1^H NMR spectrum of the starting material, 4-cyanobenzaldehyde is shown in Fig. S13). Following the two electron reduction of 0.5 M silicotungstic acid, the reduction of 4-cyanobenzaldehyde was carried out by adding the substrate to the reduced silicotungstic acid solution in stoichiometric ratios of 1 : 1 or 1 : 10 at room temperature. Substrate conversion was determined by integration of the proton signal of 4-cyanobenzaldehyde (*δ* = 10.10 ppm) in the ^1^H NMR spectra before and after reaction. Product yields were determined independently from the mass of isolated organic product obtained after extraction and solvent removal, using the molecular weight of the product to relate mass to molar amount.^20,21^ Increasing the silicotungstic acid to substrate ratio from 1 : 1 to 10 : 1 resulted in significant improvement in both conversion and yield. At a 1 : 1 ratio, substrate conversion was 12% with a product yield of 9%, whereas at a 10 : 1 ratio, substrate conversion increased to 49% and the product yield increased to 43%. In addition, a kinetic study comparing the 2-electron- and 4-electron-reduced silicotungstic acid was carried out. The substrate conversion of 4-cyanobenzaldehyde was evaluated as a function of reaction time (3, 6, 9, and 18 h) at 20 °C under a nitrogen atmosphere using a 10 : 1 molar ratio of reduced silicotungstic acid to substrate, as shown in Fig. S14. The results show a progressive increase in conversion with reaction time and consistently higher catalytic activity for the 4-electron-reduced silicotungstic acid compared with the 2-electron-reduced form.

Significantly higher conversion was observed with the 4-electron reduced silicotungstic acid. For the 2-electron reduction at a ratio of 10 : 1, 2982 coulombs were passed, resulting in a substrate conversion of 49%. In contrast, for the 4-electron reduction, 5816 coulombs were passed, leading to a significantly higher substrate conversion of 88%. These reactions were carried out using 30 mL of 0.5 M silicotungstic acid.

Table 1 summarizes the substrate conversion and isolated product yields obtained using a 10 : 1 ratio of the oxidised, two electron reduced, and four electron reduced forms of silicotungstic acid relative to substrate. Using oxidised silicotungstic acid (Entry 1) with 4-cyano benzaldehyde, no notable substrate conversion was observed, and >98% of the substrate was recovered. In contrast, significantly higher conversions and yields were achieved using the reduced forms of silicotungstic acid. Using four electron reduced silicotungstic acid resulted in yields of 81% for 4-(hydroxymethyl)benzonitrile and 23% for 4-(trifluoromethyl)benzyl alcohol, respectively (see Fig. S15 for stacked ^1^H NMR plots for 4-(trifluoromethyl)benzyl alcohol production). No side products were detected, as confirmed by ^1^H NMR and GC-MS analyses. However, compared to previous studies on the reduction of 4-cyanobenzaldehyde, the yields of 4-(hydroxymethyl)benzonitrile obtained in this study are comparatively lower. For instance, Yoswathananont *et al.* employed a continuous-flow hydrogenation system in which an aqueous solution of 4-cyanobenzaldehyde and hydrogen gas were passed through a stainless steel tube reactor packed with Pd/C under pressure.^22^ At 25 °C, this system achieved 100% conversion of 4-cyanobenzaldehyde, with the primary product being 4-(hydroxymethyl)benzonitrile, which had a yield of 72%. 4-Hydroxymethyl-benzylamine (yield 25%) and bis(4-hydroxymethyl-benzyl)amine (yield 3%) were also produced as side products. Increasing the reaction temperature to 50 °C, these authors achieved a 95% conversion of the substrate without forming any side products.^22^ It should be noted that the temperature and catalyst significantly influence the yields, which may explain the lower yields of 4-(hydroxymethyl)benzonitrile in this study. Notably, in our system, hydrogenation is mediated by an electrogenerated polyoxometalate redox mediator under aqueous conditions, without the use of a metal catalyst or external hydrogen gas, which contributes to the sustainability of the process. Additionally, as shown in the literature, the reduced silicotungstic acid used in the hydrogenation process can be recycled and reused in subsequent cycles.^7^ While the yield is lower than that reported for Pd/C continuous-flow systems, it should be noted that the two approaches are not directly comparable: our system operates without a metal catalyst or pressurised hydrogen gas, shows chemoselectivity for aldehyde reduction without forming over-reduced amine by-products, and the mediator can be electrochemically regenerated and reused. The lower yield therefore reflects the nascent stages of optimisation of the electrochemical and flow parameters rather than a fundamental limitation of the approach.

**Table 1:** Substrate conversion and isolated product yields obtained after overnight reaction (approximately 18 h) at 20 °C under a nitrogen atmosphere using a 10 : 1 ratio of 2-electron and 4-electron reduced silicotungstic acid to substrate

Entry	Substrate	Substrate recovered (%)	Substrate conversion (%)	Obtained product	Product yield (%)
1	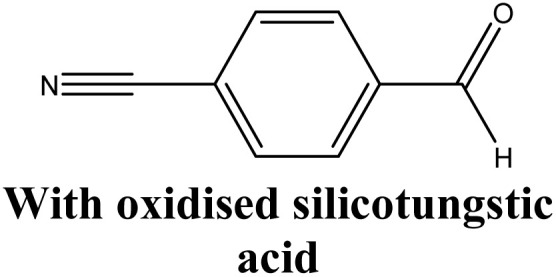	>98	—	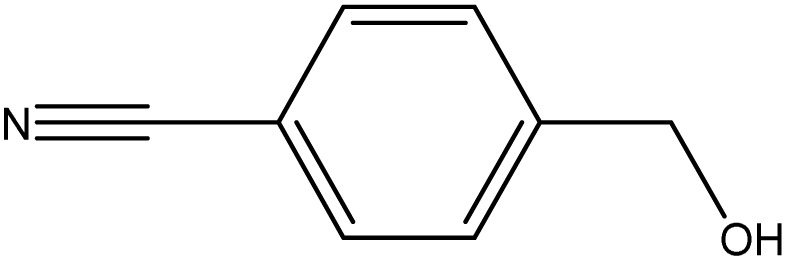	—
2	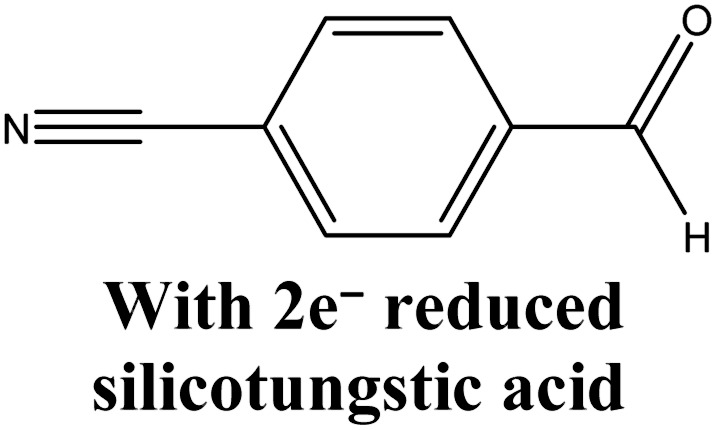	46	49	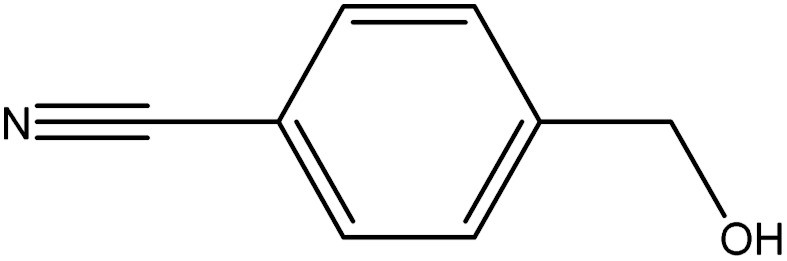	43
3	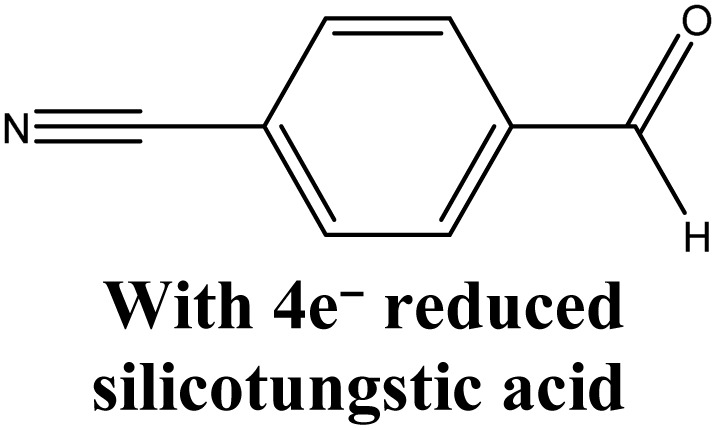	11	88	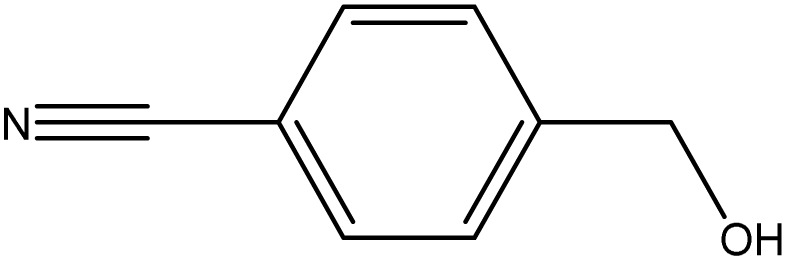	81
4	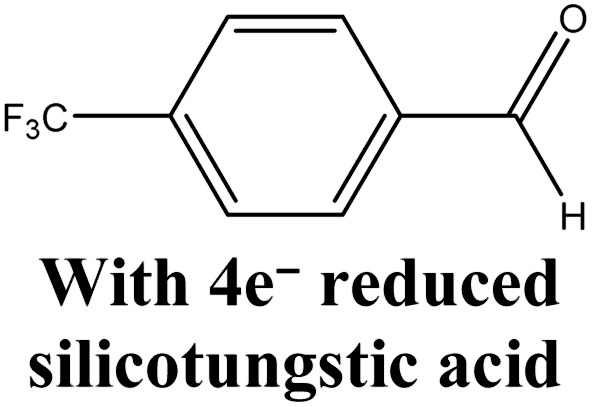	35	40	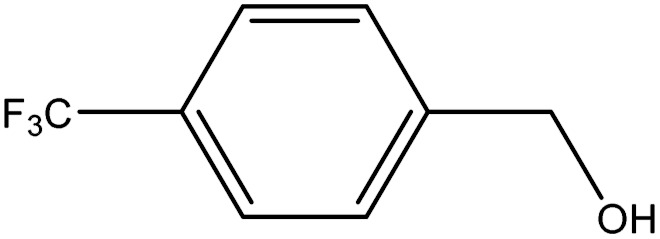	23

The reduction of both 4-cyanobenzaldehyde and 4-(trifluoromethyl)benzaldehyde to their respective products, 4-(hydroxymethyl)benzonitrile and 4-(trifluoromethyl)benzyl alcohol, was confirmed by GC-MS as shown in Fig. S16, through comparing their corresponding retention times with those of commercial substrates.

To demonstrate the reusability of the recovered silicotungstic acid, the recycled compound was subjected to electrochemical reduction to the four-electron state, as performed previously, and subsequently reused in the hydrogenation of 4-cyanobenzaldehyde. Further characterization after the third use was conducted using FTIR spectroscopy, and these results were compared with the FTIR of oxidized silicotungstic acid and its two-electron and four-electron reduced forms, as shown in Fig. S17. The characteristic water-related bands at 3473 and 1635 cm^−1^ (ref. 23), together with the preservation of the Keggin-type vibrational features, confirm that the Keggin structure remains intact throughout the reduction process, indicating no significant structural degradation. Although slight shifts and changes in the intensities of the W–O (951 cm^−1^) and W–O–W (877 cm^−1^) bands^24^ are observed in both the two-electron and four-electron reduced forms, these variations likely reflect subtle modifications in the electronic environment of the tungsten centres associated with increased electron density upon reduction. No discernible differences are observed between the product obtained from fresh electrochemically reduced silicotungstic acid and that from the electrochemically reduced and recycled silicotungstic acid, consistent with literature reports.^7,25^

For the recycling experiments, the same sample of H_4_[SiW_12_O_40_] was successfully reused up to three times, with at least 76% recovery of H_4_[SiW_12_O_40_] obtained after each cycle. The recovered catalyst was subsequently used in the reduction of 4-cyanobenzaldehyde, consistently affording 4-(hydroxymethyl)benzonitrile with a substrate conversion averaging 84% over the three reuse cycles. A recovery of H_4_[SiW_12_O_40_] of less than 100% is not unexpected after repeated extraction steps, due to its partial solubility in water and the inherent limitations in achieving quantitative recovery during recrystallization, as also reported in the literature.^7^

To investigate the reduction activity of the 4-electron-reduced silicotungstic acid toward substrates containing different electron-withdrawing groups, several aryl nitriles, aryl ketones, and aldehydes, including 1,4-dicyanobenzene, 4′-(trifluoromethyl)acetophenone, 1,2-dicyanobenzene, biphenyl-4-carboxaldehyde, and 2-naphthaldehyde, were examined. However, the reduction of these compounds was not successful, as confirmed by ^1^H NMR spectroscopy (Fig. S18–S22) and GC-MS analysis (Fig. S23–S27).

## 4 Conclusions

In summary, we have shown that optimization of electrochemical reduction parameters can allow the hitherto poorly characterized four-electron reduced form of silicotungstic acid to be produced on demand. This form has been characterized (including *via* single crystal diffraction) alongside the better-known two-electron reduced form, and the crystal structures of both these reduced forms are presented herein for the first time. The activity of both reduced forms as hydrogenation agents for the reduction of organic substrates in aqueous solution and in the absence of both hydrogen gas and external catalysts was probed, suggesting that the four-electron reduced form is the more competent hydrogenation agent. Work exploring additional deeply-reduced states of polyoxometalate redox mediators and their applications to more challenging reduction reactions are currently under investigation in our laboratories.

## Author contributions

Ahmed Aboorh: conceptualization, methodology, data curation, investigation, writing – review & editing. Zeliha Ertekin: conceptualization, methodology, investigation, writing – original draft. Sarah K. Dugmore: investigation, writing – review & editing. Claire Wilson: investigation, writing – review & editing. Mark D. Symes: conceptualization, supervision, writing – review & editing.

## Conflicts of interest

The authors declare that they have no known competing financial interests or personal relationships that could have appeared to influence the work reported in this paper.

## Supplementary Material

RA-OLF-D6RA02211A-s001

RA-OLF-D6RA02211A-s002

## Data Availability

The data underpinning this study have been deposited in the University of Glasgow's Enlighten database under accession code http://dx.doi.org/10.5525/gla.researchdata.2300. Supplementary information: the supplementary information includes data from bulk electrolysis, ultraviolet-visible absorption spectra, solid UV-vis absorption spectra, single crystal X-ray diffraction and proton nuclear magnetic resonance (^1^H NMR) spectra, gas chromatography (GC and GC-MS) and Fourier Transform Infrared (FTIR) spectroscopy. See DOI: https://doi.org/10.1039/d6ra02211a. CCDC 2500216 and 2500217 contain the supplementary crystallographic data for this paper.^26*a*,*b*^

## References

[cit1] Stergiou A. D., Symes M. D. (2022). Catal. Today.

[cit2] Falaise C. (2025). Eur. J. Inorg. Chem..

[cit3] Gumerova N. I., Rompel A. (2018). Nat. Rev. Chem..

[cit4] Smith S. P., Christian J. B. (2008). Electrochim. Acta.

[cit5] Friedl J., Holland-Cunz M. V., Cording F., Pfanschilling F. L., Wills C., McFarlane W., Schricker B., Fleck R., Wolfschmidt H., Stimming U. (2018). Energy Environ. Sci..

[cit6] Lei J., Yang J. J., Liu T., Yuan R. M., Deng D. R., Zheng M. S., Chen J. J., Cronin L., Dong Q. F. (2019). Chem.--Eur. J..

[cit7] MacDonald L., Rausch B., Symes M. D., Cronin L. (2018). Chem. Commun..

[cit8] Canny J., Liu F.-X., Hervé G. (2005). C. R. Chim..

[cit9] Launay J. P. (1976). J. Inorg. Nucl. Chem..

[cit10] Liu G.-F., Zhang S., Chen C.-J., Xing S.-M., Zhang X.-Y., Zhang Y.-J., Wu D.-Y., Li J.-F., Ren B., Chen J.-J. (2024). Chem. Mater..

[cit11] Friedl J., Pfanschilling F. L., Holland-Cunz M. V., Fleck R., Schricker B., Wolfschmidt H., Stimming U. (2019). Clean Energy.

[cit12] Keita B., Nadjo L. (1987). J. Electroanal. Chem. Interfacial Electrochem..

[cit13] Sadakane M., Steckhan E. (1998). Chem. Rev..

[cit14] Dong S., Xi X., Tian M. (1995). J. Electroanal. Chem..

[cit15] Satyananda Kishore P., Viswanathan B., Varadarajan T. (2009). Journal of Physical Chemistry.

[cit16] Misono M. , in Studies in Surface Science and Catalysis, Elsevier, 2013, 176, pp. 97–155

[cit17] Boskovic C., Sadek M., Brownlee R. T., Bond A. M., Wedd A. G. (2001). Dalton Trans..

[cit18] Wisińska N. H., Skunik-Nuckowska M., Dyjak S., Kulesza P. J. (2020). Appl. Surf. Sci..

[cit19] Klonowski P., Goloboy J. C., Uribe-Romo F. J., Sun F., Zhu L., Gandara F., Wills C., Errington R. J., Yaghi O. M., Klemperer W. G. (2014). Inorg. Chem..

[cit20] Alhathlaul N., Ertekin Z., Sproules S., Symes M. D. (2023). J. Electroanal. Chem..

[cit21] Stergiou A. D., Symes M. D. (2022). Cell Rep. Phys. Sci..

[cit22] Yoswathananont N., Nitta K., Nishiuchi Y., Sato M. (2005). Chem. Commun..

[cit23] Zhang K., Wang R. (2025). Sep. Purif. Technol..

[cit24] Fakhri H., Firooz M. H., Moteallemi A., Esrafili A., Yeganeh M., Pasalari H., Farzadkia M. (2026). Sci. Rep..

[cit25] Chen G., Li J., Yang X., Wu Y. (2006). Appl. Catal., A.

[cit26] (a) CCDC: 2500216 Experimental Crystal Structure Determination, 2026, 10.25505/fiz.icsd.cc2pxp4g

