# Supplementary material for: Preparation, characterization and hydrogenation activity of multiply-reduced silicotungstic acid
Source: RSC Adv. 2026 Jul 3;16(34):33502–10. doi: 10.1039/d6ra02211a (PMC13329783; doi:10.1039/d6ra02211a)
Supplement: RA-016-D6RA02211A-s001 [file RA-016-D6RA02211A-s001.pdf]

Supplementary Information (SI) for RSC Advances.
This journal is © The Royal Society of Chemistry 2026

Preparation, characterization and hydrogenation activity of multiply-reduced silicotungstic acid

Ahmed Aboorh,[a,b] *Zeliha Ertekin,*[a] *Sarah K. Dugmore,* [a] *Claire Wilson,* [a] *Stephen Sproules,* [a] *and Mark D. Symes**[a]

[a] *School of Chemistry, University of Glasgow, Glasgow, G12 8QQ, United Kingdom*

[b] *Department of Chemistry, College of Sciences, University of Jeddah, Jeddah 23890, Saudi Arabia*

**Email:* mark.symes@glasgow.ac.uk

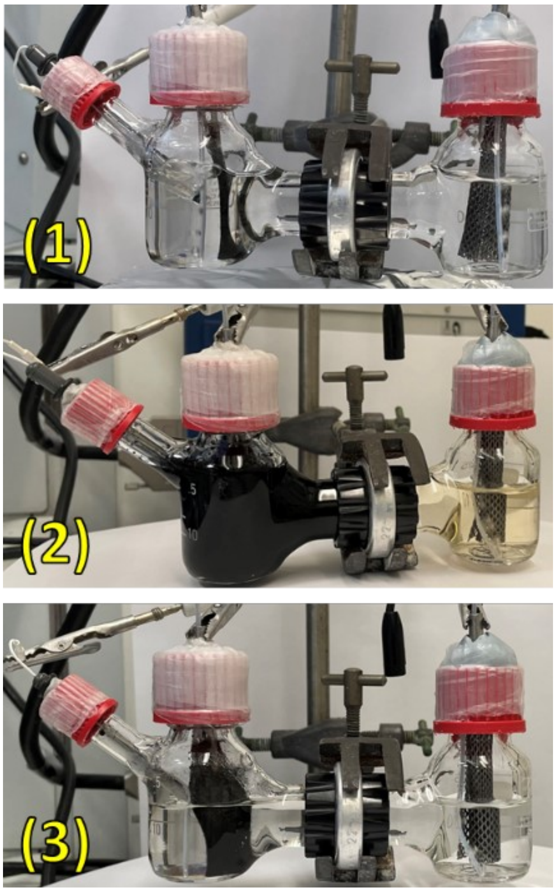

Figure S1. H-cell setup used for bulk electrolysis, illustrating three stages of the experiment: (a) the setup before the reaction, (b) after the four-electron reduction of silicotungstic acid (by applying a potential of −0.363 V *vs* NHE), and (c) following the reoxidation of silicotungstic

acid (by applying a potential of +0.757 V *vs* NHE). Note the colour change of the silicotungstic acid from colourless to blue and back to colourless.

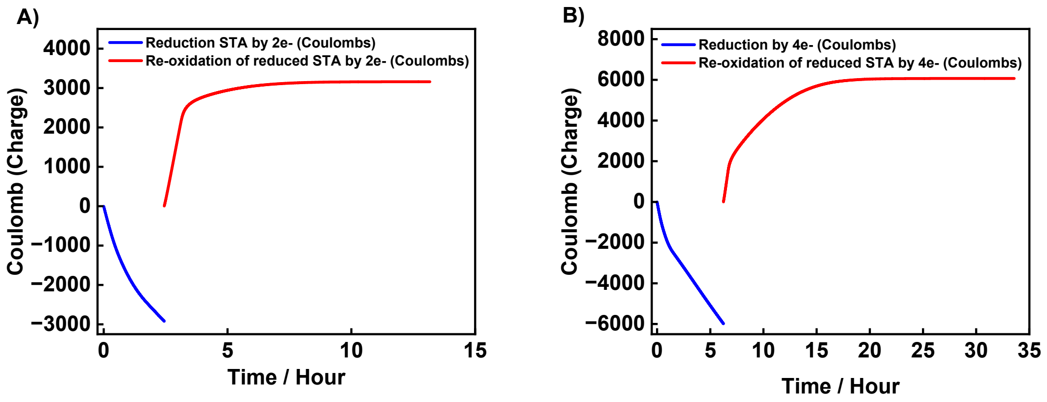

Figure S2. Charge-time plots of 2-electron (a) and 4-electron (b) reduction and reoxidation of 30 mL 0.5 M silicotungstic acid at room temperature, recorded at fixed potential of −0.36 V *vs* NHE for reduction and +0.75 V *vs* NHE for reoxidation. The experiment was conducted using a glassy carbon working electrode (0.071 cm^2), a platinum wire counter electrode, and an Ag/AgCl reference electrode in an H-cell setup. Nitrogen was bubbled continuously through the solution during the reaction.

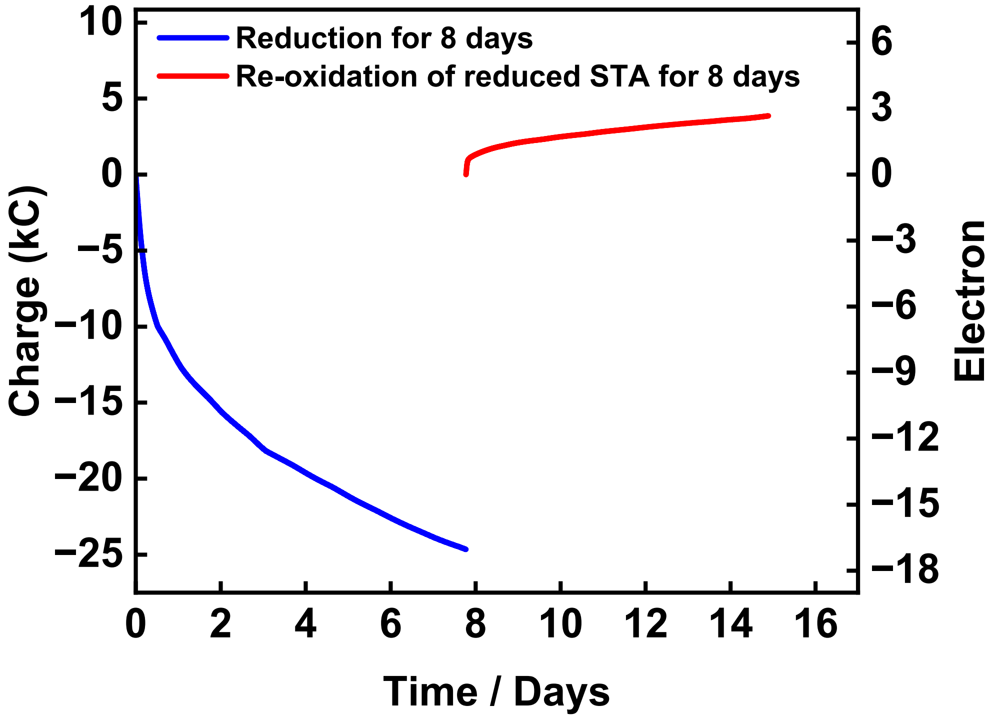

Figure S3. The long-term effects of electrochemical reduction followed by re-oxidation of 30 mL 0.5 M silicontungstic acid. The charge-time plot shows the behavior over 8 days at a fixed reduction potential of −0.36 V vs NHE, followed by oxidation at +0.76 V vs NHE, using a glassy carbon working electrode (0.071 cm^2), a platinum wire as the counter electrode and Ag/AgCl as the reference electrode in an H-cell maintained at room temperature under a nitrogen atmosphere.

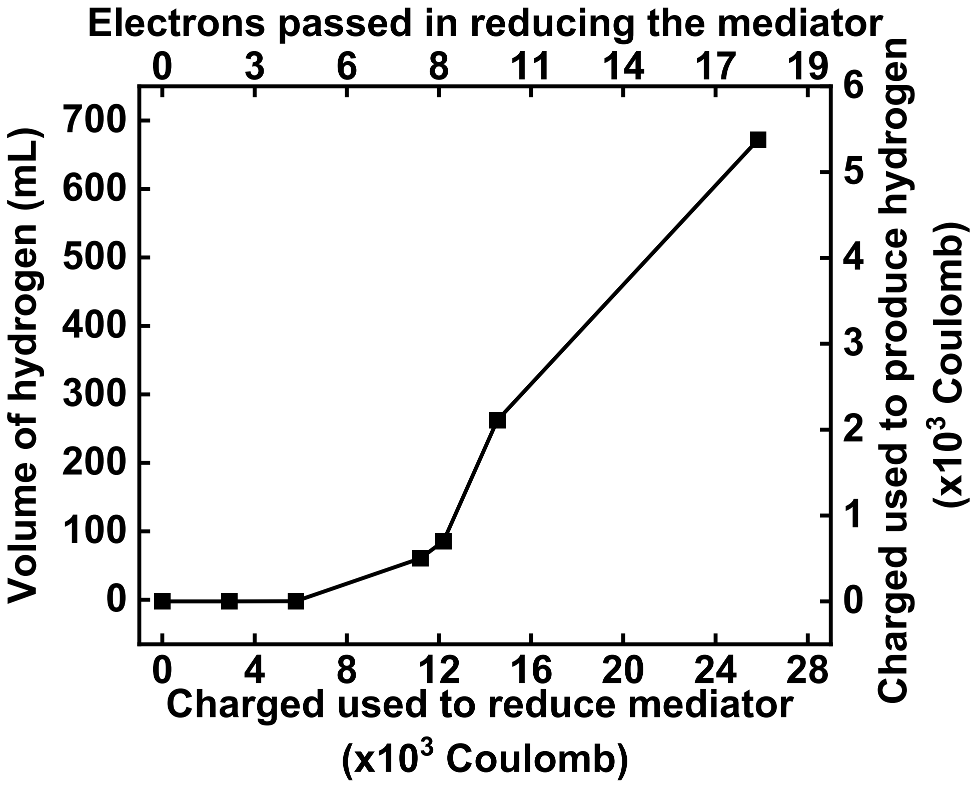

Figure S4. Dual-axis plot illustrating the relationship between the charge passed in reducing the 30 mL 0.5 M silicotungstic acid solution and the amount of hydrogen detected. The bottom x-axis represents the total charge passed during the electrochemical reduction of silicotungstic acid. The top x-axis indicates the estimated number of electrons transferred per molecule of silicotungstic acid during the initial reduction, based on the total charge passed. Meanwhile, the lefthand y-axis shows the volume of hydrogen gas detected during the reduction reaction and the righthand y-axis displays the amount of charge that is required for the production of this volume of hydrogen. Clearly ~20 kC of charge is being passed which is not going to hydrogen production.

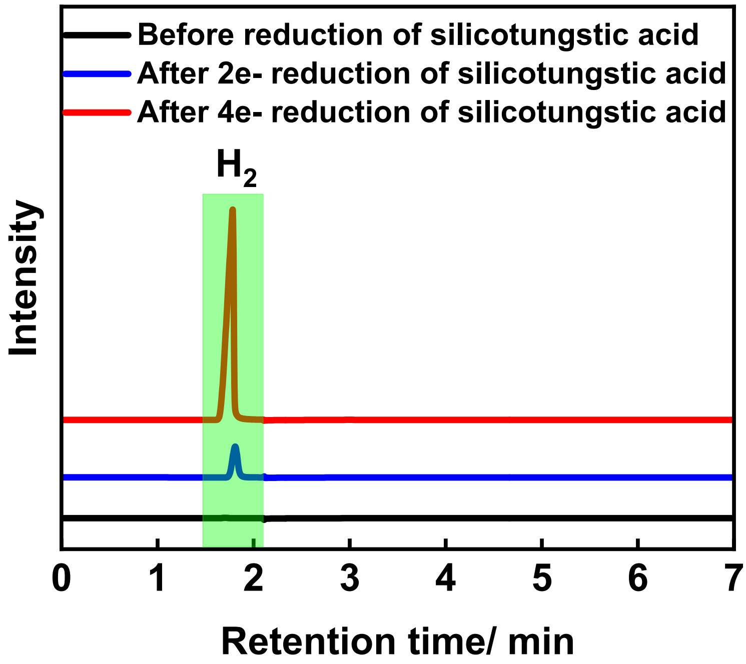

Figure S5. Gas chromatogram showing H_2 detection during the reduction of silicotungstic acid (H_2 retention time= 1.77 min).

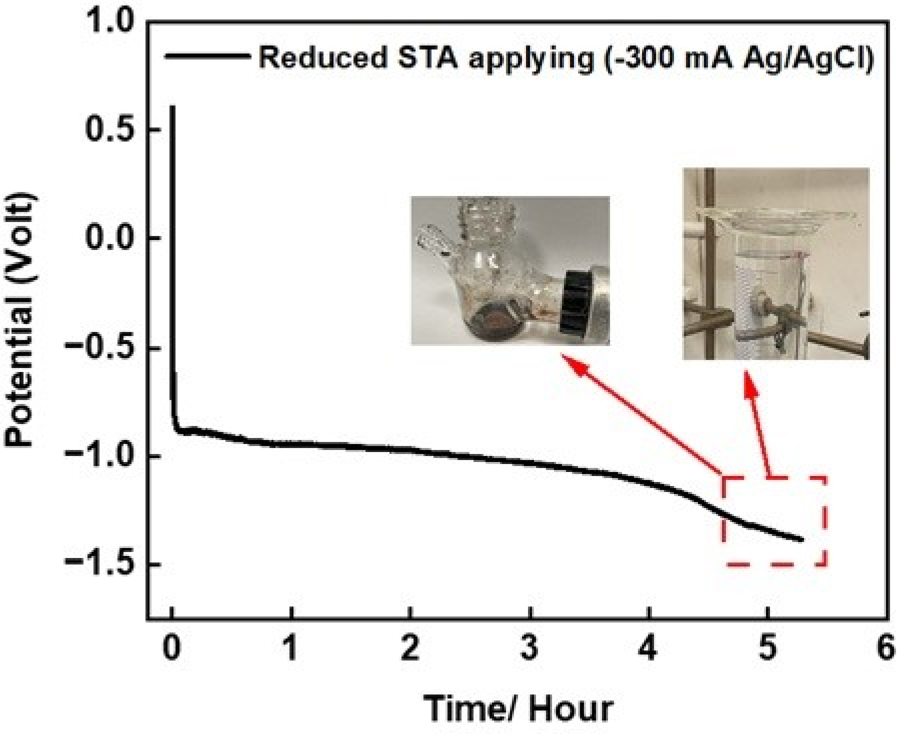

Figure S6. Charge–time curves recorded at 300 mA for 30 mL of 0.5 M silicotungstic acid. Inset: Brown-red precipitates on the H-cell cathode after the experiment, with corresponding H_2 volumes evolved.

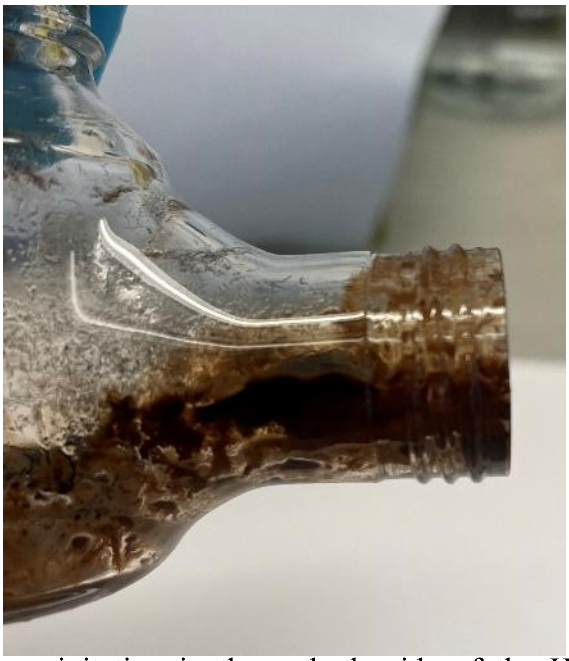

Figure S7. Red-brown precipitation in the cathode side of the H-cell after an attempted reduction of silicotungstic acid beyond 4-electrons.

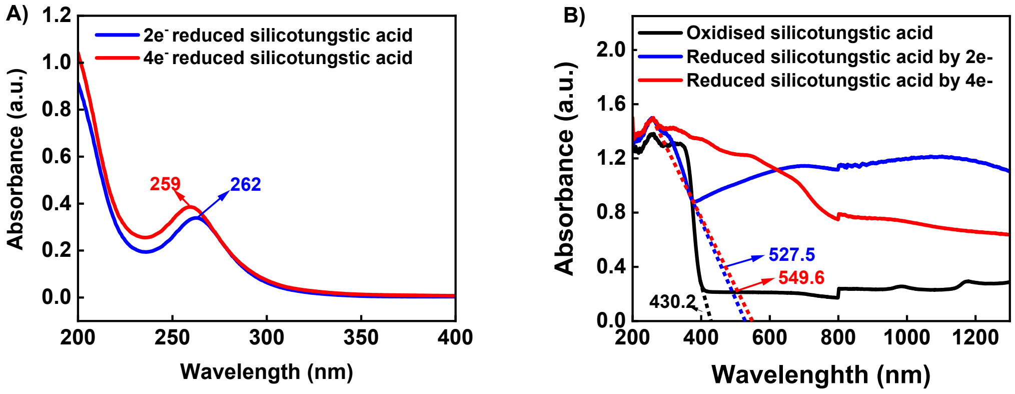

Figure S8. (a) Solution-phase ultraviolet-visible absorption spectra and **(b)** solid UV-Vis absorption spectra of silicotungstic acid reduced by 2 and 4 electrons.

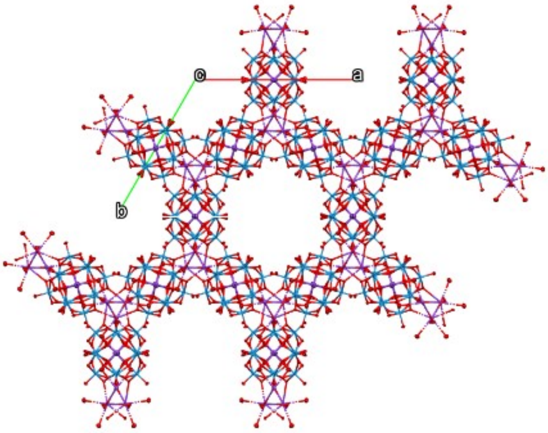

Figure S9: Ellipsoids plot of $[SiW_8^{VI}W_4^{V}O_{40}]^{8-}$ showing the water pore chanels that run

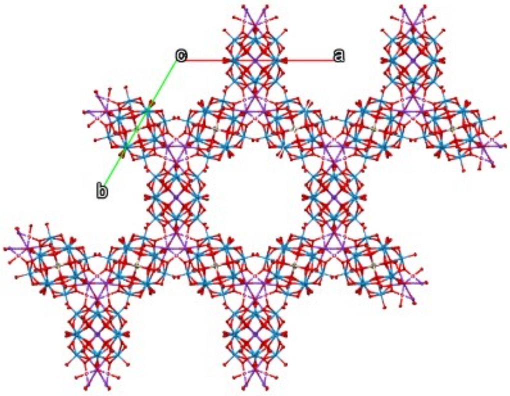

parrellel to the c-axis.

Figure S10: Ellipsoids plot of $[SiW_{10}^{VI}W_2^{V}O_{40}]^{6-}$ showing the water pore chanels that run parrellel to the c-axis.

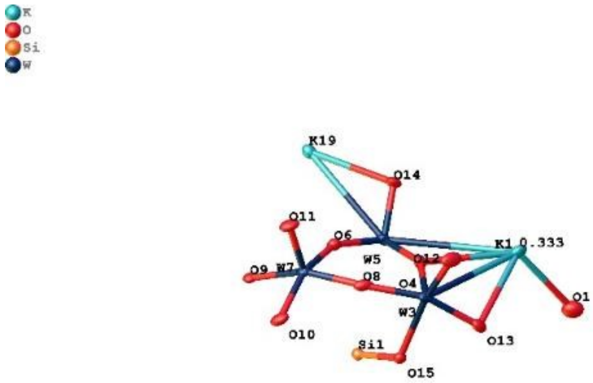

Figure S11. Ellipsoild plot of $H_2[SiW_8^{VI}W_4^{V}O_{40}]^{8-}$ showing partial K^+ occupancies, ellipsoids drawn at 50% probability.

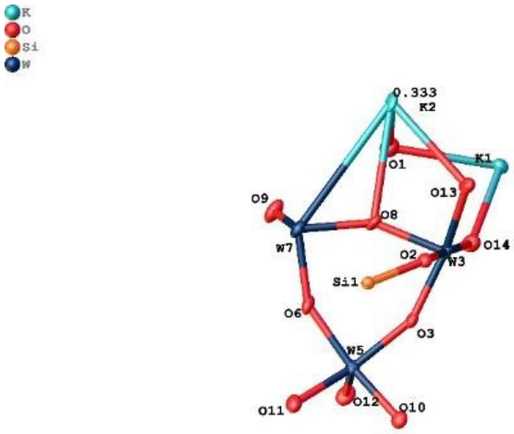

Figure S12. Ellipsoids plot of $[SiW_{10}^{VI}W_2^{V}O_{40}]^{6-}$ showing partial K^+ occupancies, ellipsoilds drawn at 50% probability.

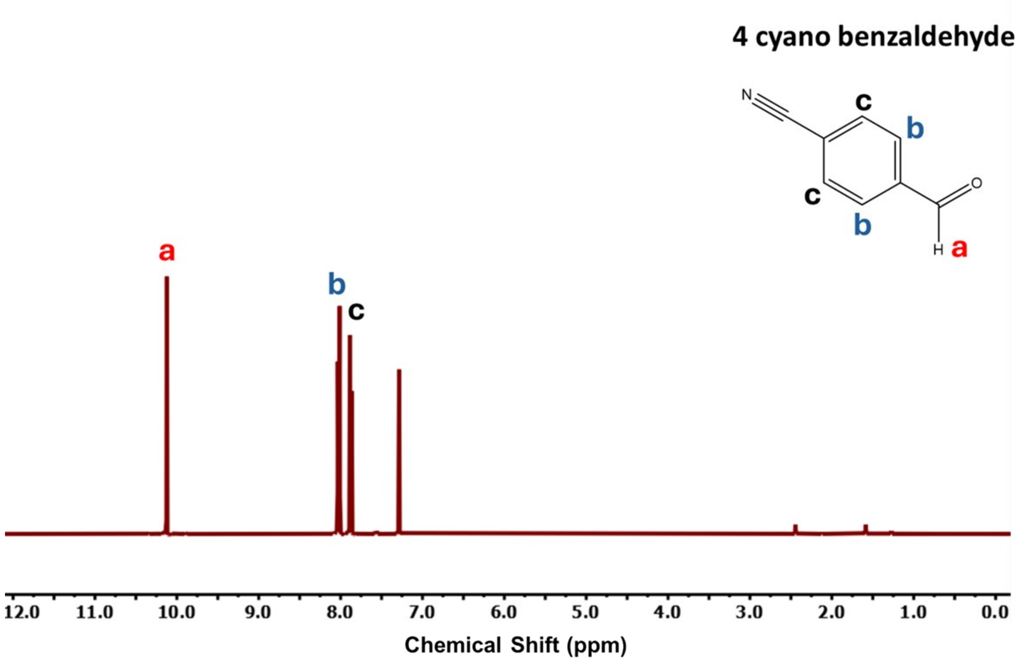

Figure S13. ^{1}H NMR spectrum of 4-cyanobenzaldehyde.

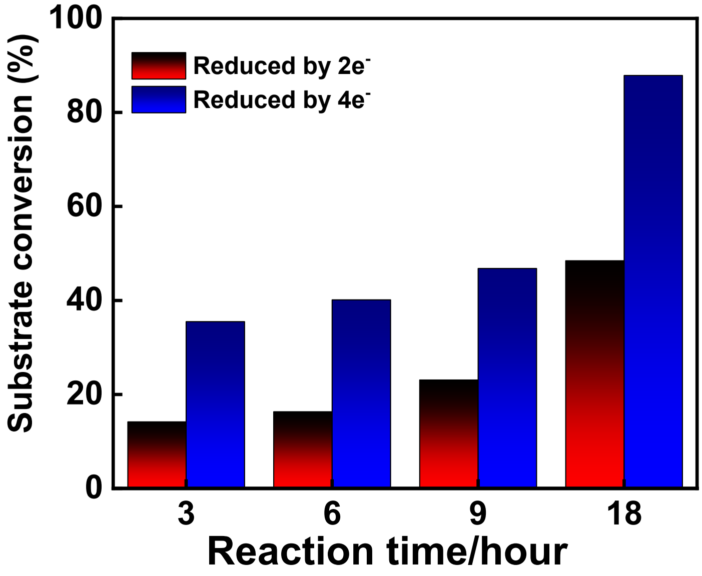

Figure S14. Substrate conversion (%) of 4-cyanobenzaldehyde as a function of reaction time (3, 6, 9, and 18 hours) at 20 °C under a nitrogen atmosphere using a 10:1 molar ratio of 2-electron and 4-electron reduced silicotungstic acid to substrate.

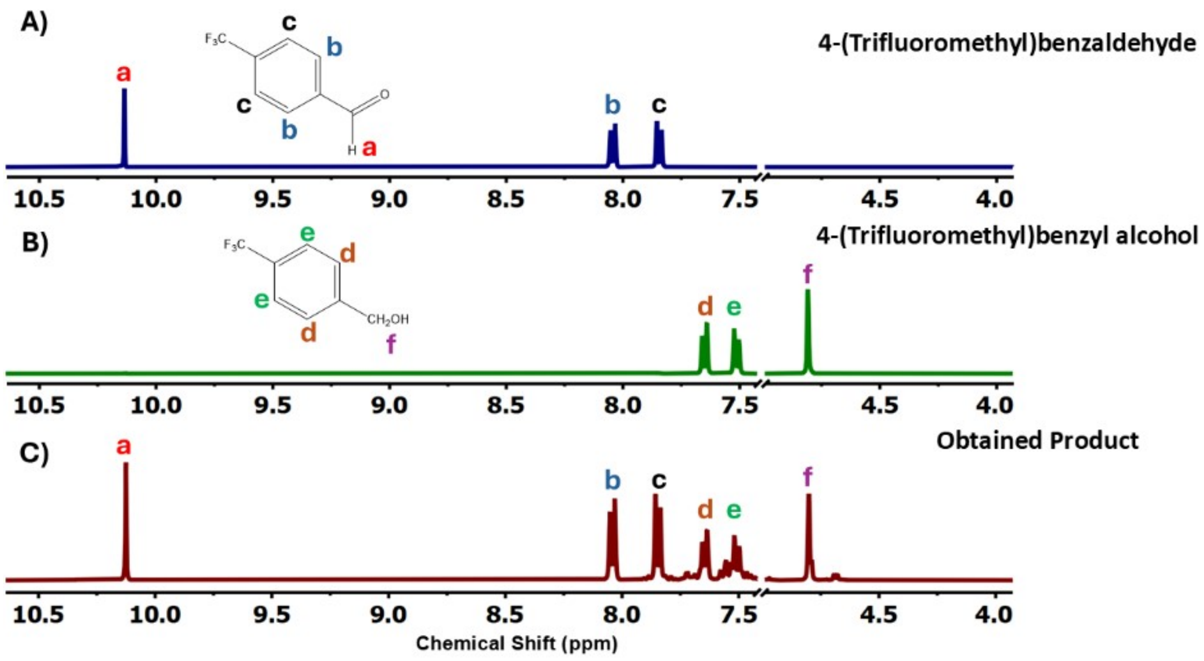

Figure S15. Stacked ^{1}H NMR spectra of **(A)** starting material 4-(trifluoromethyl)benzaldehyde, **(B)** commercial 4-(trifluoromethyl)benzyl alcohol as a standard, and **(C)** the reaction products obtained after extraction using 4-electron-reduced silicotungstic acid at a 10:1 ratio of reduced silicotungstic acid to substrate.

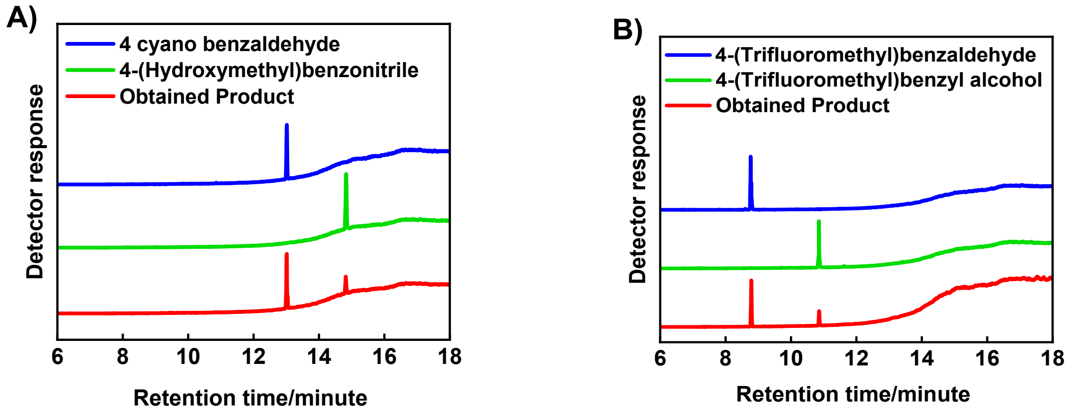

Figure S16. Stacked GC-MS analysis of **(A)** starting material 4-cyanobenzaldehyde, a commercial standard of 4-(hydroxymethyl)benzonitrile, and the obtained product containing both 4-cyanobenzaldehyde and 4-(hydroxymethyl)benzonitrile, and **(B)** starting material 4-(trifluoromethyl)benzaldehyde, a commercial standard of 4-(trifluoromethyl)benzyl alcohol, and the obtained product containing both 4-(trifluoromethyl)benzaldehyde and 4-(hydroxymethyl)benzonitrile , from the reaction of 4-electron reduced silicotungstic acid with these substrates.

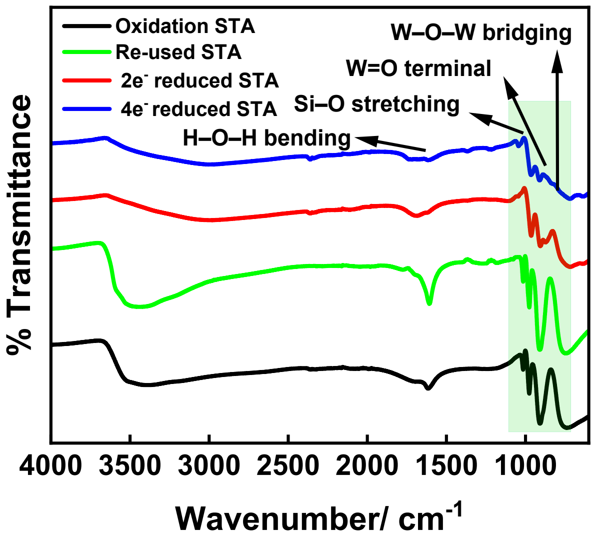

Figure S17. FTIR spectra of oxidized silicotungstic acid (black line), two-electron reduced silicotungstic acid (blue line), four-electron reduced silicotungstic acid (red line), and recycled silicotungstic acid after three reuse cycles (green line).

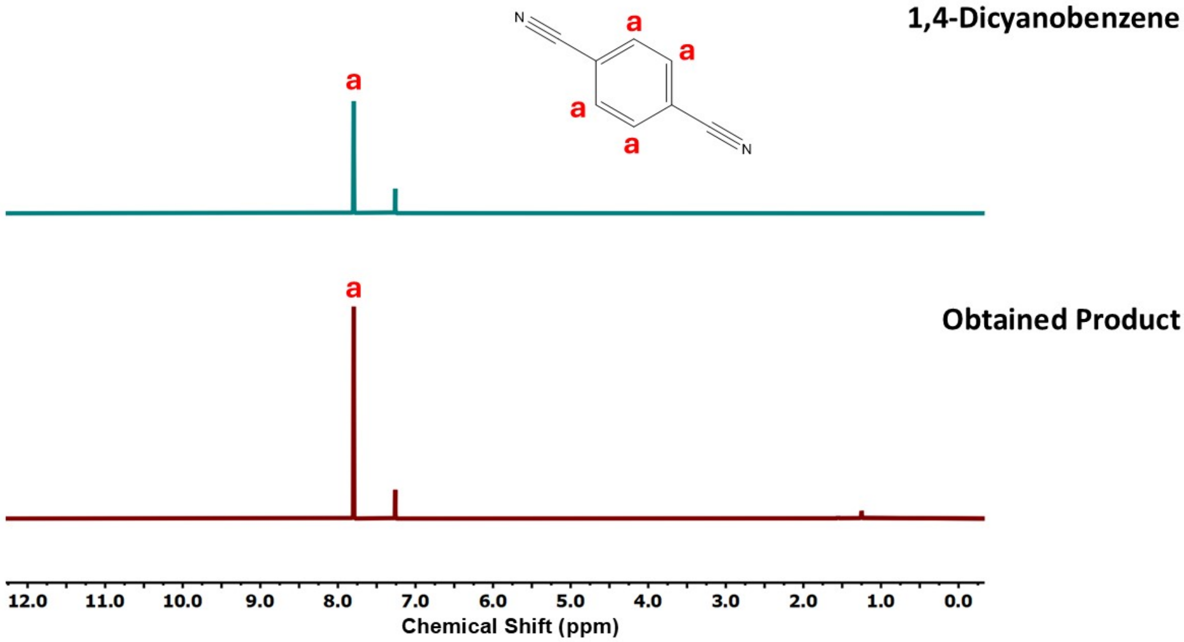

Figure S18. Stacked ^{1}H NMR spectra (400 MHz, $CDCl_3$) of 1,4-dicyanobenzene and the product obtained after attempted reaction with 4-electron reduced silicotungstic acid.

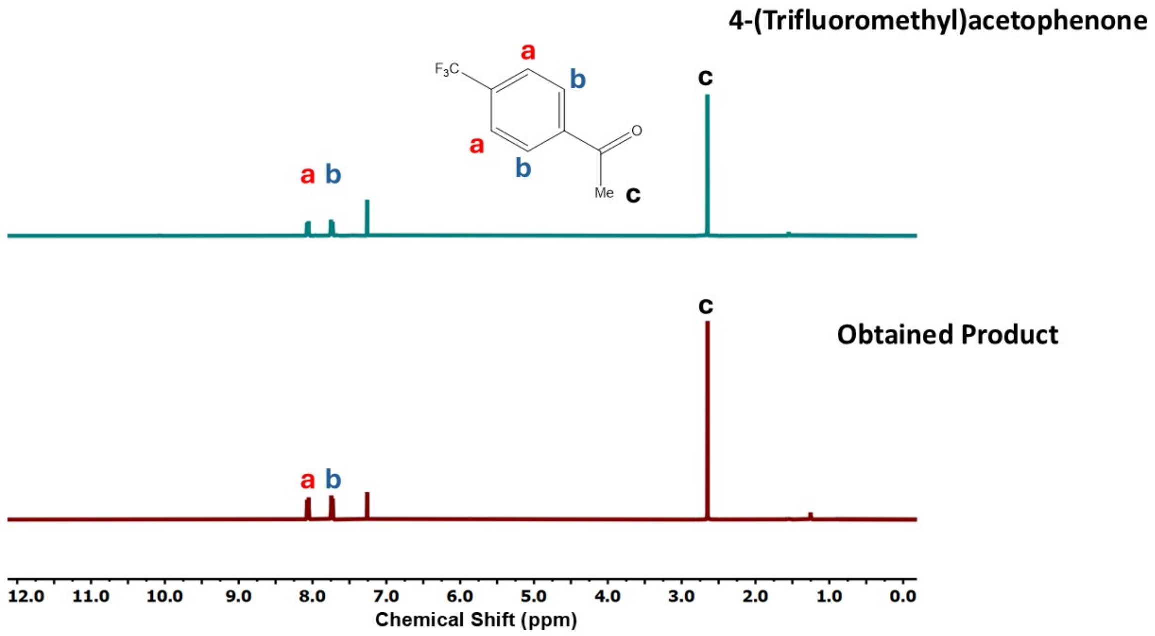

Figure S19. Stacked ^{1}H NMR spectra (400 MHz, $CDCl_3$) of (4-(trifluoromethyl)acetophenone and the product obtained after attempted reaction with 4-electron reduced silicotungstic acid.

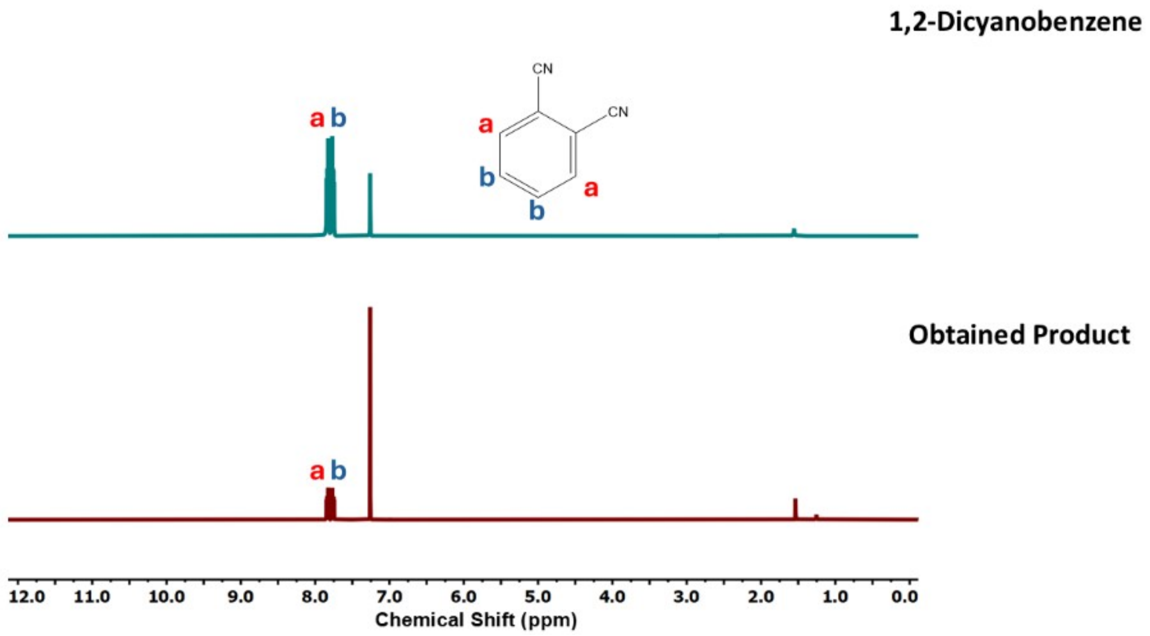

Figure S20. Stacked ^{1}H NMR spectra (400 MHz, $CDCl_3$) of 1,2-dicyanobenzene and the product obtained after attempted reaction with 4-electron reduced silicotungstic acid.

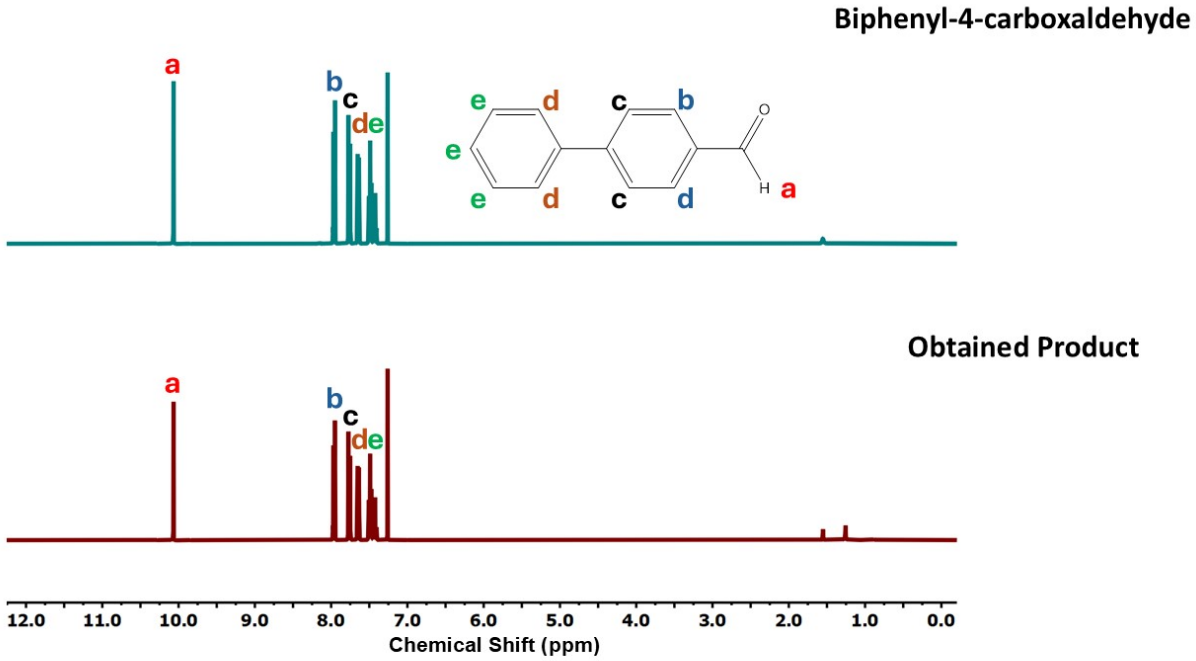

Figure S21. Stacked ^{1}H NMR spectra (400 MHz, $CDCl_3$) of biphenyl-4-carboxaldehyde and the product obtained after attempted reaction with 4-electron reduced silicotungstic acid.

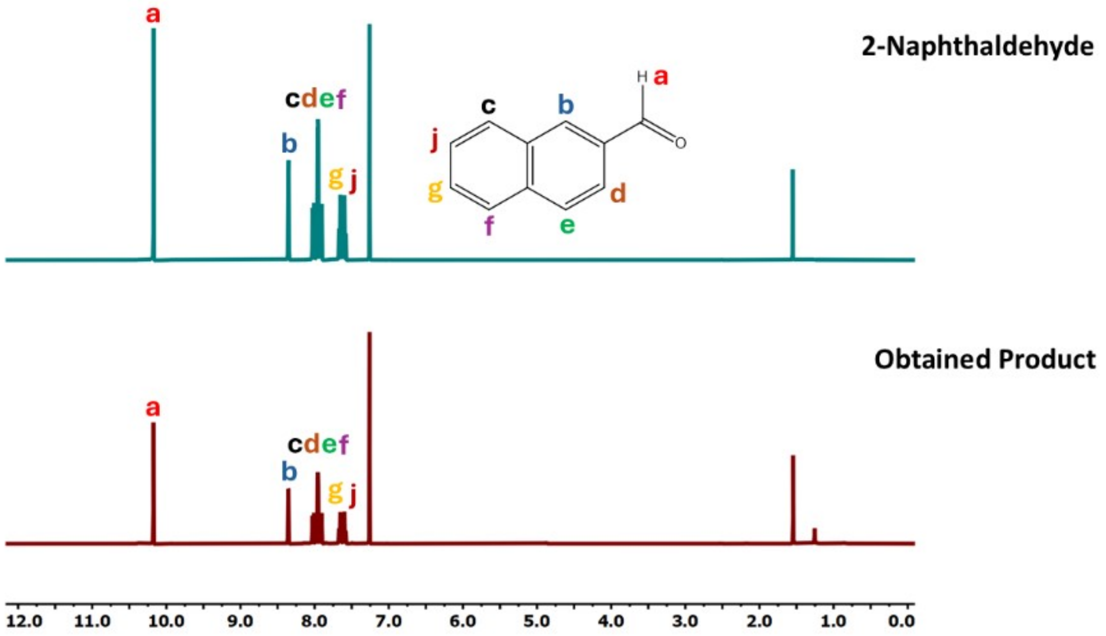

Figure S22. Stacked ^{1}H NMR spectra (400 MHz, $CDCl_3$) of 2-naphthaldehyde and the product obtained after attempted reaction with 4-electron reduced silicotungstic acid.

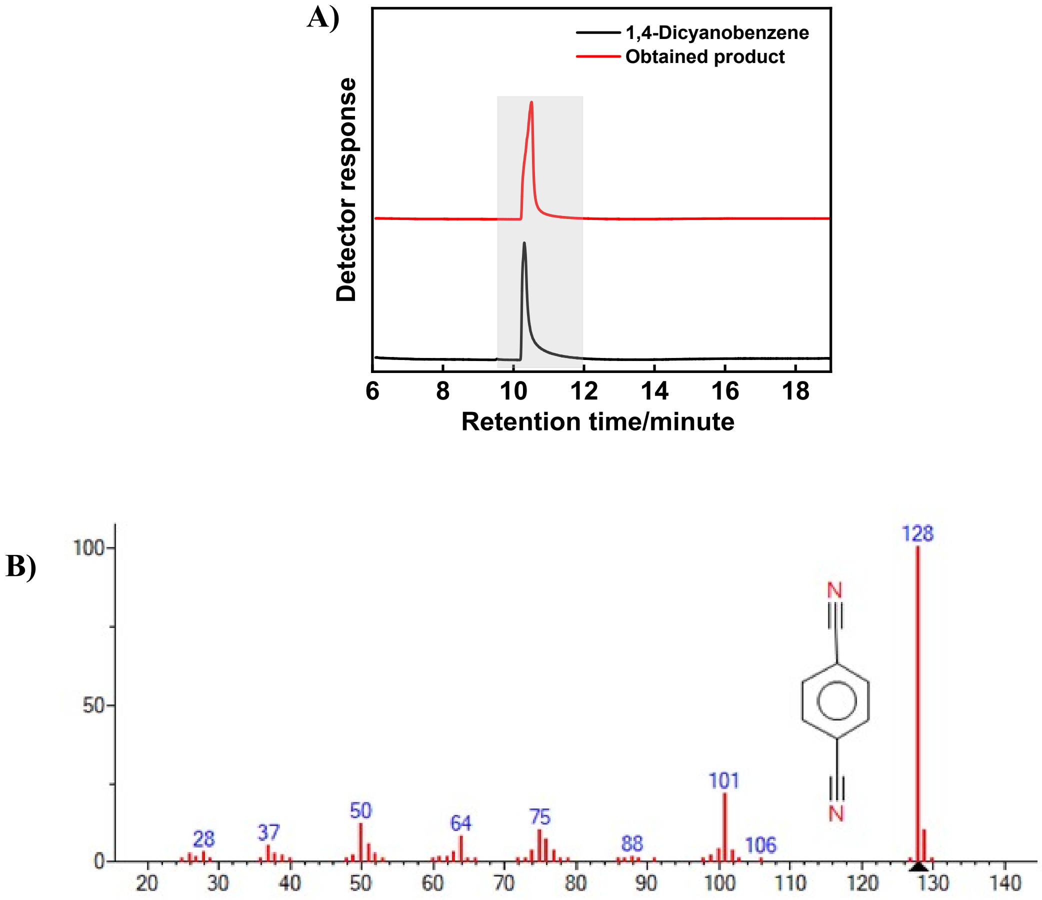

Figure S23. (A) Stacked gas chromatography analysis of the starting material 1,4-dicyanobenzene (black line) and the product obtained after attempted reaction with 4-electron reduced silicotungstic acid (red line). **(B)** Mass spectrum showing molecular structure matched with the NIST library at a retention time of 10.37 min.

A)

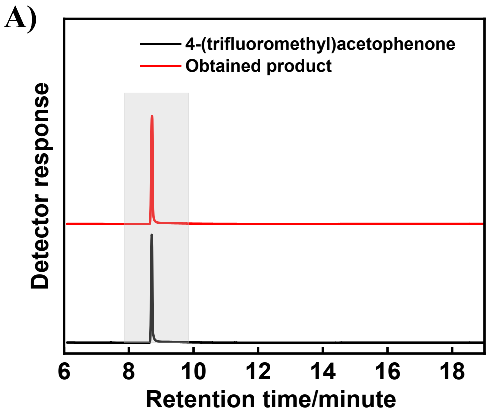

B)

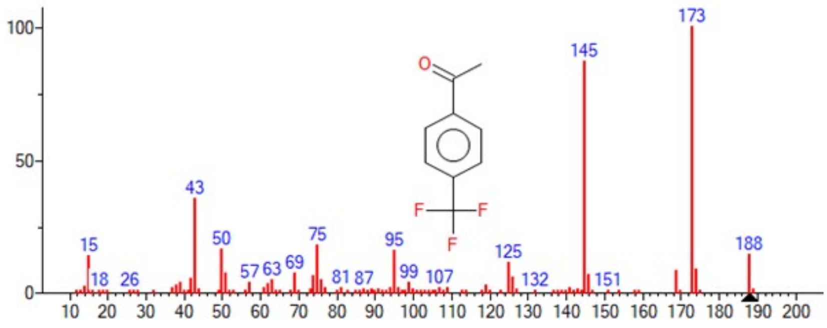

Figure S24. (A) Stacked gas chromatography analysis of the starting material 4-(trifluoromethyl)acetophenone (black line) and the product obtained after attempted reaction with 4-electron reduced silicotungstic acid (red line). **(B)** Mass spectrum showing molecular structure matched with the NIST library at a retention time of 8.710 min.

A)

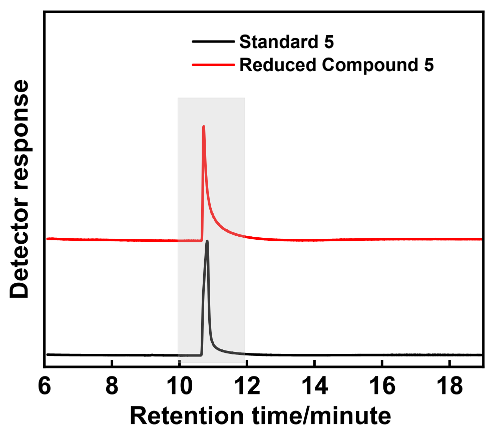

B)

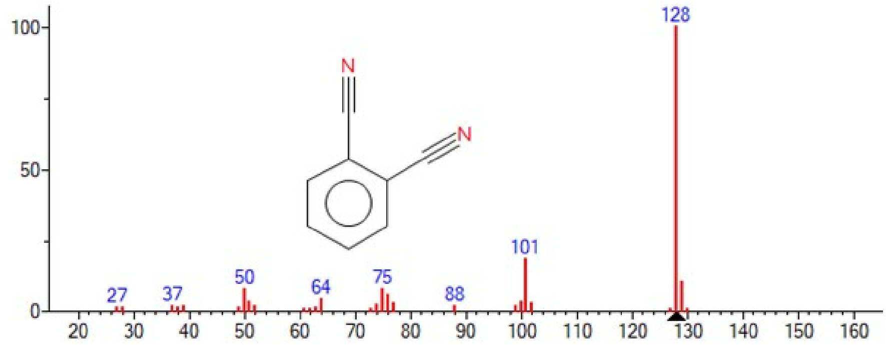

Figure S25. (A) Stacked gas chromatography analysis of the starting material 1,2-Dicyanobenzene (black line) and the product obtained after attempted reaction with 4-electron reduced silicotungstic acid (red line). **(B)** Mass spectrum showing molecular structure matched with the NIST library at a retention time of 10.82 min.

A)

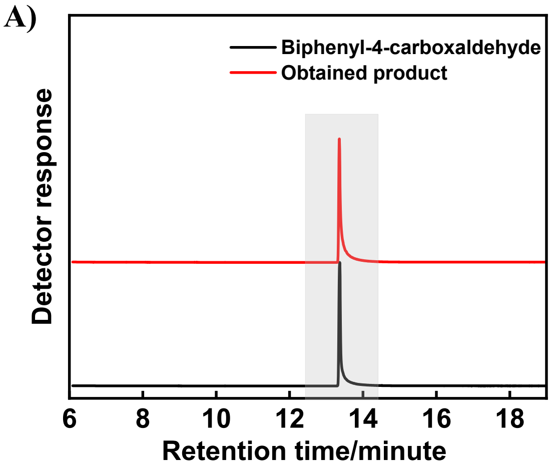

B)

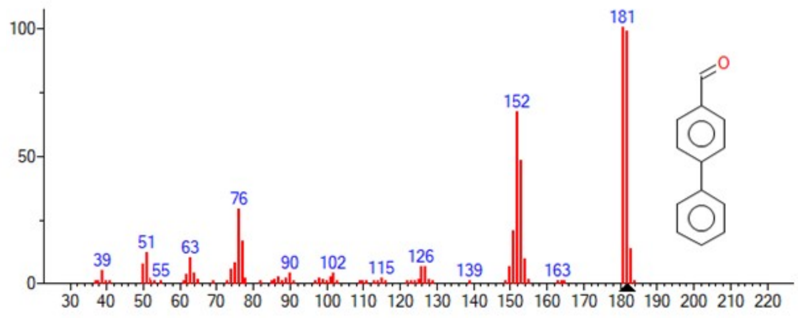

Figure S26. (A) Stacked gas chromatography analysis of the starting material Biphenyl-4-carboxaldehyde (black line) and the product obtained after attempted reaction with 4-electron reduced silicotungstic acid (red line). **(B)** Mass spectrum showing molecular structure matched with the NIST library at a retention time of 13.36 min.

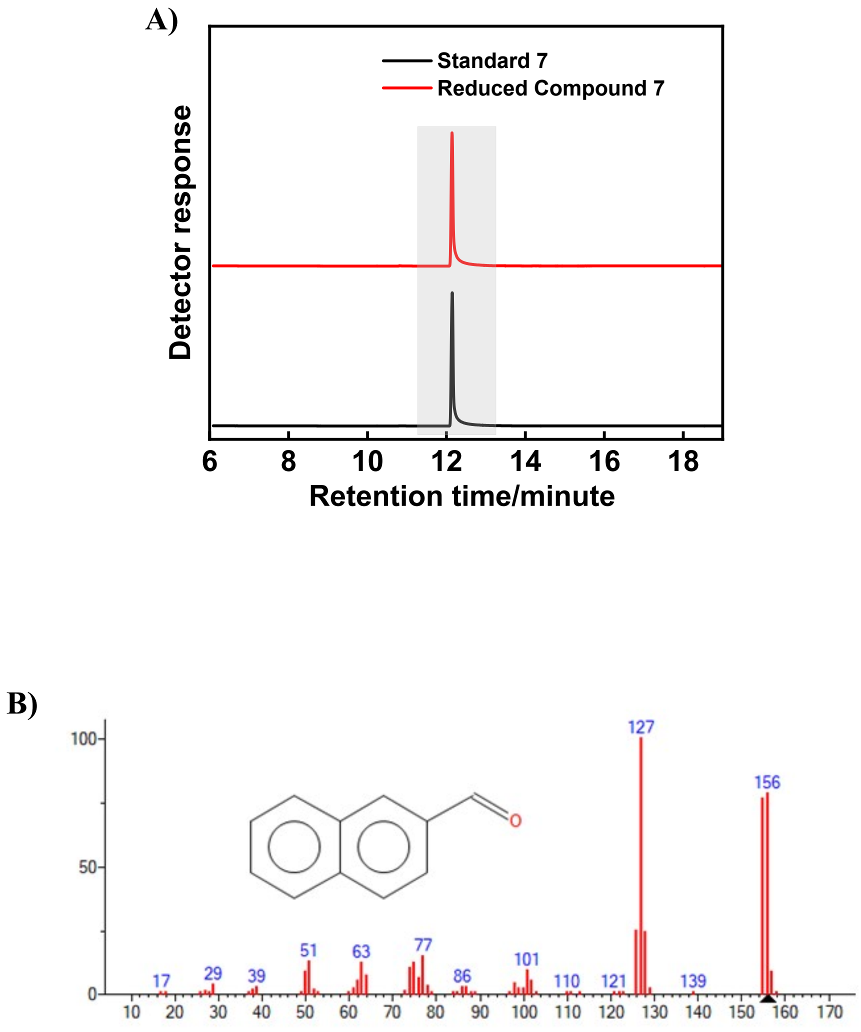

Figure S27. (A) Stacked gas chromatography analysis of the starting material 2-Naphthaldehyde (black line) and the product obtained after attempted reaction with 4-electron reduced silicotungstic acid (red line). **(B)** Mass spectrum showing molecular structure matched with the NIST library at a retention time of 12.14 min.

Table S1. Comparison of crystallographic data and structural parameters for oxidized silicotungstic acid[1] and its two electron and four electron reduced silicotungstate anions.

Parameter	Oxidized $K_4[SiW_{12}O_{40}]^{4-}$	Oxidized $K_4[SiW_{12}O_{40}]^{4-}$	2e⁻ reduced $K_3H_3[SiW_8^{VI}W_4^{V}O_{40}]^{6-}$	4e⁻ reduced $K_3H_5[SiW_8^{VI}W_4^{V}O_{40}]^{8-}$
**Space group**	$P6_2\,2\,2$	$P6_2\,2\,2$	$P6_2\,2\,2$	$P6_2\,2\,2$
a/b (**Å**)	19.1618	18.9987 (5)	18.9922(2)	18.9540(3)
c (**Å**)	12.5124	12.57(4)	12.5148(1)	12.5283(2)
Volume (**Å^3**)	3978.72	3929.7(19)	3909.35(9)	3897.84(14)
**Temperature (K)**	293	150	150	150
Mean (W–O) (**Å**)	1.92 (est.)	-	1.93 (est.)	1.94 (est.)
Avg. W Bonnd Valence	+5.85	+5.85	+6.09	+6.12
K^+ occupancy per Keggin ion	4	-	≈ 2.7	≈ 2.7
**Solvent mask (e⁻ per ASU)**	-*	-	42 (≈ 4.2 H_2O)	45 (≈ 4.5 H_2O)

*The oxidised structure published [1] contains ~7 water molecules per Keggin Ion.

Single Crystal X-ray Diffraction Data

4e⁻ reduced STA (CCDC 2500216)

Table S2. Crystal data for the 4e- reduced STA

$K_{2.667}O_{44}SiW_{12}$·0.333	D_x = 3.912 Mg m^{-3}
M_r = 3060.63	Mo *K*a radiation, l = 0.71073 Å
Hexagonal, $P6_222$	Cell parameters from 17353 reflections
a = 18.9540 (3) Å	q = 2.5–35.8°
c = 12.5283 (2) Å	m = 26.79 mm^{-1}
V = 3897.84 (14) Å^3	*T* = 150 K
Z = 3	Block, black
F(000) = 3948	0.08 × 0.06 × 0.04 mm

Table S3. Data collection for the 4e⁻ reduced STA

XtaLAB Synergy R, HyPix-Arc 150 diffractometer	3168 reflections with *I* > 2s(*I*)
Detector resolution: 10.0000 pixels mm^{-1}	R_{int} = 0.041
w scans	q_{max} = 28.3°, q_{min} = 2.5°
Absorption correction: multi-scan *CrysAlis PRO* 1.171.43.90 (Rigaku Oxford Diffraction, 2023) Empirical absorption correction using spherical harmonics,	*h* = -25®22

implemented in SCALE3 ABSPACK scaling algorithm.	
T_{min} = 0.606, T_{max} = 1.000	k = -24®25
32721 measured reflections	l = -16®16
3241 independent reflections	

Table S4. Refinement details for the 4e- reduced STA

Refinement on F^2	12 restraints
Least-squares matrix: full	$w = 1/[s^2(F_o^2) + (0.0112P)^2 + 7.1204P]$ where $P = (F_o^2 + 2F_c^2)/3$
$R[F^2 > 2s(F^2)]$ = 0.015	$(D/s)_{max}$ = 0.002
$wR(F^2)$ = 0.030	$D\rho_{max}$ = 0.56 e Å^{-3}
S = 1.04	$D\rho_{min}$ = -0.65 e Å^{-3}
3241 reflections	Absolute structure: Flack x determined using 1265 quotients [(I+)-(I-)]/[(I+)+(I-)] (Parsons, Flack and Wagner, Acta Cryst. B69 (2013) 249-259).
140 parameters	Absolute structure parameter: -0.006 (8)

2e⁻ Reduced STA (CCDC 2500217)

Table S5. Crystal data for the 2e- reduced STA

$K_{2.667}O_{44}SiW_{12}\cdot 0.333[]$	D_x = 3.900 Mg m^{-3}
M_r = 3060.63	Mo Ka radiation, l = 0.71073 Å
Hexagonal, $P6_222$	Cell parameters from 23746 reflections
a = 18.9922 (2) Å	q = 2.7–37.4°
c = 12.5148 (1) Å	m = 26.71 mm^{-1}
V = 3909.35 (9) Å^3	T = 150 K
Z = 3	Needle, metallic dark blue
F(000) = 3948	0.43 × 0.08 × 0.04 mm

Table S6. Data collection for the 2e- reduced STA

XtaLAB Synergy R, HyPix-Arc 150 diffractometer	3243 independent reflections
Radiation source: micro-focus sealed X-ray tube, Mova (Mo) X-ray Source	3163 reflections with $I > 2s(I)$
Mirror monochromator	R_{int} = 0.040
Detector resolution: 10.0000 pixels mm^{-1}	q_{max} = 28.3°, q_{min} = 2.5°
w scans	h = -24®25
Absorption correction: multi-scan *CrysAlis PRO* 1.171.43.90 (Rigaku Oxford Diffraction, 2023) Empirical absorption correction using spherical harmonics,	k = -25®25

implemented in SCALE3 ABSPACK scaling algorithm.	
T_{min} = 0.349, T_{max} = 1.000	l = -15®16
36374 measured reflections	

Table S7. Refinement details for the 2e⁻ reduced STA

Refinement on F^2	Primary atom site location: dual
Least-squares matrix: full	$w = 1/[s^2(F_o^2) + (0.0153P)^2 + 4.0569P]$ where $P = (F_o^2 + 2F_c^2)/3$
$R[F^2 > 2s(F^2)]$ = 0.014	$(D/s)_{max}$ = 0.001
$wR(F^2)$ = 0.031	$D\rho_{max}$ = 0.95 e Å^{-3}
S = 1.03	$D\rho_{min}$ = -0.80 e Å^{-3}
3243 reflections	Absolute structure: Flack x determined using 1263 quotients [(I+)-(I-)]/[(I+)+(I-)] (Parsons, Flack and Wagner, Acta Cryst. B69 (2013) 249-259).
140 parameters	Absolute structure parameter: -0.001 (6)
48 restraints	

References

1. P. Klonowski, J. C. Goloboy, F. J. Uribe-Romo, F. Sun, L. Zhu, F. Gandara, C. Wills, R. J. Errington, O. M. Yaghi and W. G. Klemperer, *Inorganic Chemistry*, 2014, **53**, 13239–13246.
